# Quality parameters for a multimodal EEG/EMG/kinematic brain-computer interface (BCI) aiming to suppress neurological tremor in upper limbs

**DOI:** 10.12688/f1000research.2-282.v2

**Published:** 2014-04-30

**Authors:** Giuliana Grimaldi, Mario Manto, Yassin Jdaoudi

**Affiliations:** 1 Unité d’Etude du Mouvement, Université Libre de Bruxelles, Erasme, Bruxelles, 1070, Belgium; 2 Fonds de la Recherche Scientifique, Université Libre de Bruxelles, Bruxelles, 1070, Belgium

## Abstract

Tremor is the most common movement disorder encountered during daily neurological practice. Tremor in the upper limbs causes functional disability and social inconvenience, impairing daily life activities. The response of tremor to pharmacotherapy is variable. Therefore, a combination of drugs is often required. Surgery is considered when the response to medications is not sufficient. However, about one third of patients are refractory to current treatments. New bioengineering therapies are emerging as possible alternatives. Our study was carried out in the framework of the European project “Tremor” (ICT-2007-224051). The main purpose of this challenging project was to develop and validate a new treatment for upper limb tremor based on the combination of functional electrical stimulation (FES; which has been shown to reduce upper limb tremor) with a brain-computer interface (BCI). A BCI-driven detection of voluntary movement is used to trigger FES in a closed-loop approach. Neurological tremor is detected using a matrix of EMG electrodes and inertial sensors embedded in a wearable textile. The identification of the intentionality of movement is a critical aspect to optimize this complex system. We propose a multimodal detection of the intentionality of movement by fusing signals from EEG, EMG and kinematic sensors (gyroscopes and accelerometry). Parameters of prediction of movement are extracted in order to provide global prediction plots and trigger FES properly. In particular, quality parameters (QPs) for the EEG signals, corticomuscular coherence and event-related desynchronization/synchronization (ERD/ERS) parameters are combined in an original algorithm which takes into account the refractoriness/responsiveness of tremor. A simulation study of the relationship between the threshold of ERD/ERS of artificial EEG traces and the QPs is also provided. Very interestingly, values of QPs were much greater than those obtained for the corticomuscular module alone.

## Introduction

Tremor is the most common movement disorder encountered during daily practice ^1^. It causes functional disability and social inconvenience, disturbing daily life activities. Its incidence and prevalence increase with ageing ^1^. The response of tremor to pharmacotherapy is variable and a combination of drugs is often required after a few years of therapy. Neurosurgical procedures are considered when the clinical response is not sufficient or when the patient becomes refractory. However, a number of patients do not respond to current therapies. Therefore novel strategies are being developed. New bioengineering therapies are now emerging as viable solutions ^2^. In particular, recent studies aim to develop and validate a new treatment for upper limb tremor based on the combination of functional electrical stimulation (FES) with a brain-computer interface (BCI) ^2– 3^. The main goal is to set up a semi-automatic procedure to reduce/stop upper limb tremor, with a selective cancellation of tremor oscillations while preserving natural voluntary movement. The intentionality of movement is tracked by the BCI, in order to trigger FES in the upper limbs. Such concepts open new doors for the treatment of numerous neurological disorders affecting the upper limbs.

We describe a multimodal detection of the intentionality of movement by fusing signals from EEG, EMG and kinematic sensors (in particular gyroscopes and accelerometers). A kinematic module is applied purely for analyzing tremor, but also finds a specific application for the early detection of movement in patients presenting with a rest tremor - a tremor occurring while in a rest position. Indeed, it has been reported that patients presenting a rest tremor show a decrement of the rest tremor before voluntary movement onset ^4^. This phenomenon might be induced by a cortico-cerebellar activation during voluntary movements ^5^. Why the use of a multimodal detection of the intentionality of movement? Although the potential for BCIs in neurological disorders is huge, the applicability of current BCI systems has been limited by several factors ^6^. One of them is the poor performance of BCIs based on EEG analysis only (also due to: inter-individual differences in the detectability of movement-related EEG-activity; differences in the way BCI users can voluntary modify their brain activity; and the fact that brain atrophy and neuroplastic changes occurring in patients affected with movement disorders makes it difficult to generalize EEG markers). Therefore, this multimodal processing is assumed to add accuracy in the prediction of movements, thus improving the effectiveness of the system.

## Materials and methods

**Figure 1.**
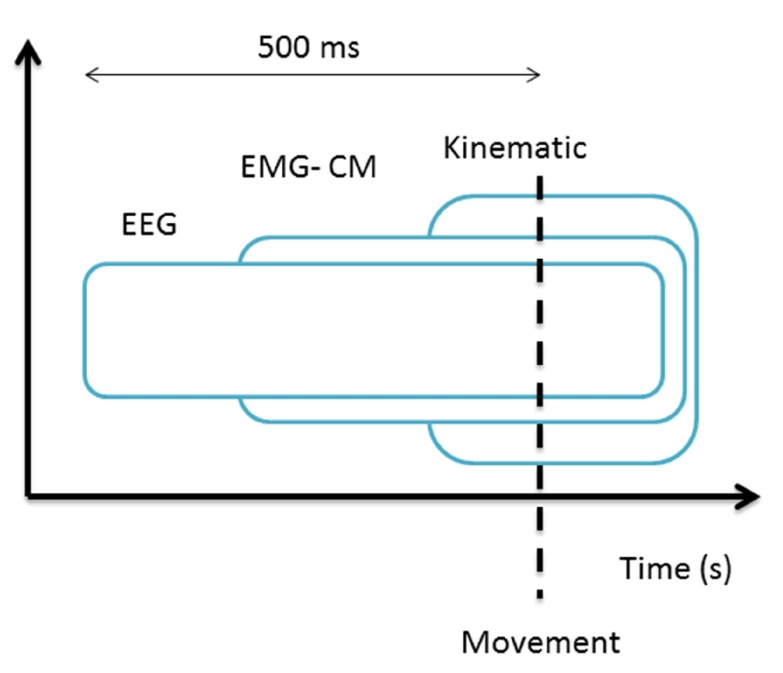
Expected prediction order. Different modules are used to predict a voluntary movement: EEG, EMG, Corticomuscular (CM) and a Kinematic module. Notice that movement might be predicted by the kinematic module, as happened in the case of rest tremor decreasing before a voluntary movement.

Figure 1 gives a schematic glance at the multimodal approach. From each module, acting during different time-windows (EEG, kinematic and corticomuscular (described in detail in sections C–F) quality parameters (QPs) for the detection of the intentionality of movement or for the early detection of movement are extracted. QPs were calculated for each movement executed by the patients (one run contains several movements; see section B). These QPs are also considered as probabilities of stimulation, given their potential application in a tremor suppression system based on BCI-trigged FES (see also section F).

### A. Description of patients

Acquisition of data was carried out on 4 neurological patients exhibiting a bilateral upper limb tremor (combinations of rest, postural and/or kinetic tremor), following approval of the Ethical Committee of ULB – Hôpital Erasme ( Table 1). All the patients were followed at the Erasme Hospital and gave their written informed consent to participate in the study. Patients were affected by: Parkinsonism of vascular origin (n=1), Parkinson’s disease (n=1), essential tremor (n=1) and post-traumatic brain injury (n=1). Male/female ratio was 3:1. Mean age of the patients was 62±20 years. The patients were all right-handed and presented with upper limb tremor of grade 1 to 3/4. The ADL-T24 score range was 3–20/24 ^7^. The Schwab and England ADL score ranged from 50 to 80% ^8^.

**Table 1.** Description of patients.

Subject	Sex	Age	Disease	Rest tremor	Kinetic tremor	ADL-T24 score	Schwab and England ADL score
001	M	83	Parkinsonism of vascular origin	1/4	¼	3	70%
007	M	53	Parkinson’s Disease	2/4	¼	9	80%
009	F	75	Essential tremor	0/4	¼	4	80%
012	M	38	Post-traumatic brain injury	0/4	¼	20	50%

### B. Experimental set-up

The patients were comfortably seated and performed sequences of "finger-to-nose" movements cued by acoustic signals. The patients kept their eyes open. The dominant arm was studied. The finger-to-nose task consists of touching the nose with the index finger, keeping the index finger on the nose for about one second and then putting it back onto the thigh (starting position). Patients were told to keep the most relaxed attitude. After hearing an acoustic signal, they prepared themselves for the execution of movement by mental imagery of the movement. During a single run, the task was repeated about 10 times. Patients were first trained in order to perform the task correctly. Each patient executed a maximum of 6 runs. The nomenclature used for the recorded files–as reported in figures- is “pppFNnn” standing for patient’s code, task executed ("Finger-to-nose") and run number, respectively.

Patients were equipped with:

(i) IMU sensors (inertial measurement units: tri-axial gyroscopes, accelerometers, magnetometers). Two IMUs were located on the anterior face of the upper limb at about 4 cm above and below the elbow, respectively. Sensors were attached with tape.

(ii) a conventional EEG cap with the following location of EEG electrodes (international 10–20 system): FC3, FCz, FC4, C5, C3, C1, CZ, C2, C4, C6, CP3, CPZ, and CP4 (POz: ground; linked ear-lobes: reference). Artifacts were minimized by restraining head movements, keeping the jaw and face relaxed and by avoiding swallowing or blinking during the recordings. Artifact rejection was applied by visual inspection of traces. EEG signals were sampled at 256 Hz (re-sampling at 1000 Hz for synchronization purposes) and band-pass filtered at 0.5–60 Hz.

(iii) EMG multi-array electrodes (arrays of 16 electrodes) located on the flexor carpi radialis (FCR), extensor carpi radialis (ECR), biceps and triceps muscles. EMG data were sampled at 1 KHz.

### C. Movement detection

**Figure 2.**
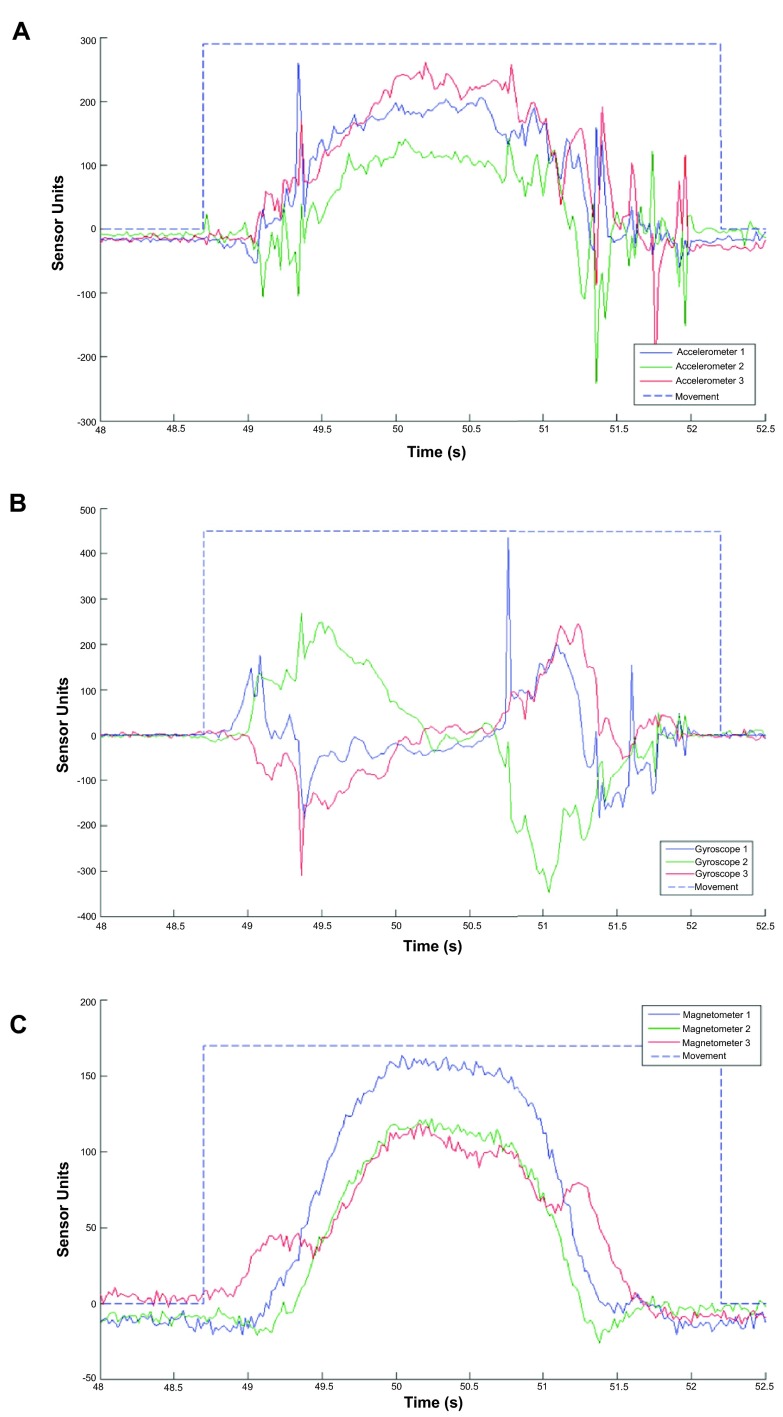
Movement detection based on signal processing. **A**: signal processing of an accelerometer; **B**: of a gyroscope; and **C**: of a magnetometer (triaxial sensors: axis x, y, and z). The dotted line corresponds to a voluntary movement.

The main goal of this module is to identify the beginning and the end of a movement in the time domain. In order to build a “movement window”, the signal from the magnetometer (which provides a very clean signal) is processed first. The delay generated with magnetometers is then corrected with accelerometer and gyroscope signals. This results in an “extended movement window” within a time frame of 500 ms before the “basic movement window” generated by the magnetometer signal alone ( Figure 2). Each variation (in accelerometer and gyroscope channels) larger than the standard deviation channel will extend the basic ‘movement window’ until the detected variation. This module, in the multimodal strategy, will be used by all other modules to determine whether a context is well predicting a movement or a false positive is occurring.

### D. EEG module

Cortical activation occurring during the preparation of movement is detected by the EEG module thanks to a method based on the event-related desynchronization/synchronization (ERD/ERS) phenomenon ^7, 9, 10^. We extracted a QP for the detection of intentionality of movement ^11^ by considering: (i) the changes in the β²/α and β/α ratio (representing bursts of β-γ frequencies) during the pre-movement period; (ii) an appropriate threshold indicating which peaks of ratios are actually followed by a movement (and therefore may be considered as a predictor of movement); (iii) the number of movements executed.

Upsampled EEG data were processed with a Hamming window of 256 samples, using an overlap of 250 in the time domain. Spectrograms were computed at the frequencies from 1 Hz to 40 Hz with the Goertzel algorithm using a short time Fourier Transform (STFT) ^12^. A one-sided power spectral density (PSD) matrix was then obtained with the following formula:

$$P=\frac{2{\left|S(i,j)\right|}^{2}}{Fs\sum_{n=1}^{L}{\left|w(n)\right|}^{2}}\;\;\;\;\;(1)$$

Where P contains the PSD of each segment for the frequency range 1–40 Hz, w(n) denotes the Hamming window function and Fs is the sampling frequency (1000 Hz).

Three time intervals were studied: pre-movement period, movement period, post-movement period. The pre-movement period (lasting 2 seconds) was defined according to the acoustic order given to the patients and the detection of the beginning of movement via the gyroscopes, by considering 2 seconds back from the point of detection of the beginning of movement. We decided to use a period of 2000 msec based on the available literature which considers that 2 seconds encompasses the preparation phase at the cortical level ^13^.

The α, β and γ frequency bands were compared by calculating β/α and β²/α ratios. PSD in a β-γ frequency band was divided by the PSD in the α frequency band:

$$ratio(t)=10\log_{10}\frac{\sum \beta_{f}^{n}(t)}{\sum \alpha_{f'}^{}(t)}\;\;\;\;\;(2)$$

Where n = {1,2} depends on the ratio considered (squared or not), f is the interval of β-γ frequencies (e.g.: from 26 to 33 Hz), and f’ is the interval of α frequency (e.g.: from 8 to 10 Hz).

To extract these sub-bands, the following intervals in the α, β and γ frequency bands were first studied: 8–12 Hz (8 Hz, 9 Hz, 10 Hz, 11 Hz, 12 Hz), 8–10 Hz, 10–12 Hz, 13–40 Hz, 13–26 Hz, 26–40 Hz, 13–20 Hz, 20–26 Hz, 26–33 Hz, 33–40 Hz, 13–16 Hz, 16–20 Hz, 20–23 Hz, 23–26 Hz, 26–30 Hz, 30–33 Hz, 33–40 Hz. Therefore, each β-γ interval was compared with the α intervals. A total amount of 105 pairs of intervals were thus analyzed. By applying (2) to the EEG power spectra from all the EEG channels and the successive runs, we obtained ratiograms which are spectrogram-like representations of EEG activities on the skull. The peaks (β/α and β²/α ratios) higher than a defined threshold were considered as indicators of a potential voluntary movement ^11^, given that they represent the detection of the cortical motor preparation of the movement ^14, 15^. To determine the occurrence of false positive results, the number of movements detected was added. EEG QP is the geometric mean of the probability of movement (true positive stimulations) and the percentage of movements predicted ^11^.

### E. Corticomuscular module (EEG-EMG)

A low-pass filter at 30 Hz was applied to EMG data. Corticomuscular coherence is a function of frequency (with values between 0 and 1) and indicates the degree of correlation between the two signals ^10^. The Welch's averaged modified periodogram method was used to compute the magnitude squared coherence of an EEG channel and an EMG electrode along the frequency subbands ^7, 12, 16^. More than 800 possible EEG/EMG combinations (n=832) were tested for the cortico-muscular coherence analysis:

$$C_{xy}(f)=\frac{{\left|P_{xy}(f)\right|}^{2}}{P_{xx}(f)P_{yy}(f)}\;\;\;\;\;(3)$$

Where *P _xx_* and *P _yy_* are the PSDs and *P _xy_* is the cross-spectral density, *f* is the frequency and *C _xy_* is the magnitude squared coherence. The signal was first segmented in 200 ms squared windows. Each window was then processed with an FFT (length 512, window of 8 samples, and overlap of 6).

### F. Kinematic module

As mentioned above, the kinematic module was applied both to characterize tremor and for the early detection of movement in patients presenting with a rest tremor. Up-sampled gyroscope signals (from 50 Hz to 1 KHz) were processed with a Hamming window of 256 samples, an overlap of 250 in the time domain. The spectrogram was computed at the frequencies from 1 Hz to 20 Hz with the Goertzel algorithm using a STFT. A one-sided PSD matrix was then obtained with the following equation:

$$P=\frac{2{\left|S(i,j)\right|}^{2}}{Fs\sum_{n=1}^{L}{\left|w(n)\right|}^{2}}\;\;\;\;\;(4)$$

Where *P* contains the PSD of each segment for the frequency range 1–20 Hz, *w(n)* denotes the Hamming window function and *Fs* is the sampling frequency (1000 Hz).

The prediction of the movement was based on two major features of the pre-movement period:

   - a 200 ms gap in the spectrum corresponding to a temporary dramatic decrease of the tremor

   - a rise of low frequencies.

The rise of low frequencies was used for a mathematical modelling which considered:

   - the ratio of high frequencies (7–20 Hz) divided by low frequencies (0–7 Hz)

   - the max PSD in the low-frequency band over time.

A threshold was then applied and the prediction was based upon the following algorithm:

*If (ratio(t) > T and maxPSD(t) > T) then*

       
*Potential movement predicted*

*Else*

       
*No movement detected*

*End*

Where t is the time and T stands for threshold and is defined as the standard deviation of the ratio and the maxPSD.

The kinematic QP extracted is the following:

$QP\text{​}=\sqrt{p\times n}$ Where *p* is the probability of movement and *n* the number of movements predicted, as previously described for the EEG QP ^11^. A QP can be derived for one axis (X or Y or Z) or from several axes combined. Probability of movement *p* represents a key signal for the BCI-triggered delivery of FES to launch the muscle stimulation. Thus, this parameter is also named “probability of stimulation”. QP is an index of prediction of movement, while the “probability of stimulation/of movement” is the accuracy of this index, corresponding to the true positives.

## Results

### A. EEG module

**Figure 3.**
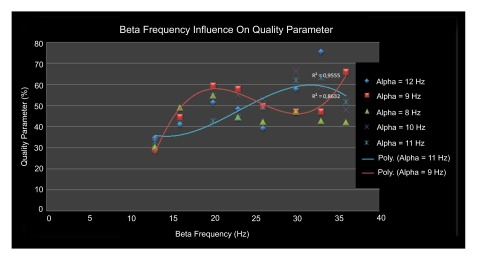
Influence of beta band frequency on EEG quality parameter (QP). Two peaks can be identified for a beta frequency of 20 Hz and 35 Hz. Polynomial fitting (order 3) for an alpha frequency of 11 Hz (blue; R² = 0.9555) and 9 Hz (red; R² = 0.8632).

The EEG QP allowed the prediction of the voluntary movement with a probability between 70% and 90%. The mean QP was 82±12% (median = 83.5%) for the β/α ratio and 79.5±10.4% (median = 80%) for the β²/α ratio. We found no significant difference between the QP calculated from β/α ratio and β²/α ratio (p = 0.502) ^11^. The highest QPs were found when the selected sub-band of frequency included the 30–35 Hz ( Figure 3). A sub-band of interest was more difficult to identify for the α band. However, the entire α band and its sub-bands never provided low values of QP. In terms of QP distribution on the scalp, the central areas of the brain showed the highest values of QPs ^11^. The highest probability to predict efficiently the intention of the upper limb movement corresponded to the contralateral central area of the brain.

### B. Corticomuscular module

**Figure 4.**
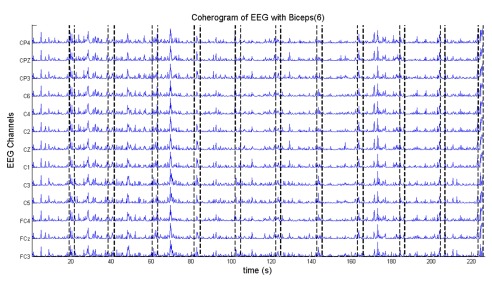
Coherogram showing the evolution of coherence over time during voluntary movements of the upper limb (EEG channels-central area of the brain-correlated to an EMG electrode of the biceps muscle). The black vertical dotted lines correspond to the detected movements.

**Figure 5.**
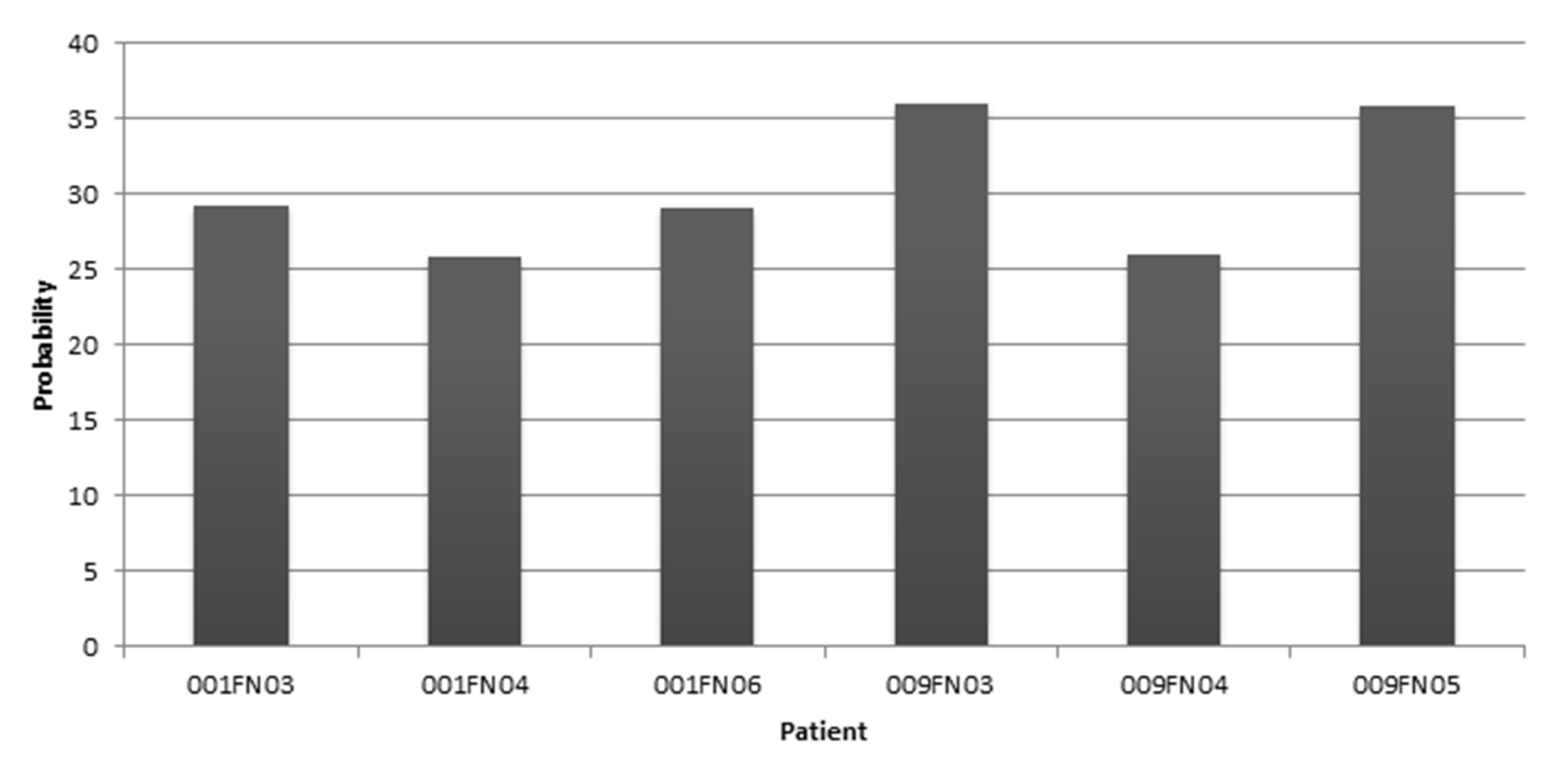
Probability of stimulation. Results of corticomuscular coherence from patient 001 (presenting a Parkinsonism of vascular origin) and from patient 009 (presenting essential tremor). Three trials were analyzed for each patient.

By applying the process described in the *Corticomuscular module* section of the Materials and methods for each EEG channel compared to an EMG electrode, we obtained a graphical representation of the corticomuscular coherence (coherogram, Figure 4). A coherogram can be designed in different ways: either combining all EEG channels with one EMG electrode or associating all electrodes of an EMG device with one EEG channel. Here the first option was chosen because the possibility of a practical implementation of this approach in clinical applications is greater. Statistics for one coherogram channel were obtained by applying a threshold equal to the standard deviation. The same process was then applied on all channels. Data from two patients (3 trials for each patient) were analyzed in-depth (see additional Data Set). Probabilities of stimulation were extracted. For example, the coherence probability reached a maximum of 35.97% for patient 009FN03 (ECR muscle). Figure 5 shows the maximum values from all the possible EEG/EMG channels combinations. These patients exhibited reproducible low values for the cortico-muscular coherence, by contrast to reproducible high values for the other QPs. This highlights the importance of our multimodal approach.

### C. Kinematic module

**Figure 6.**
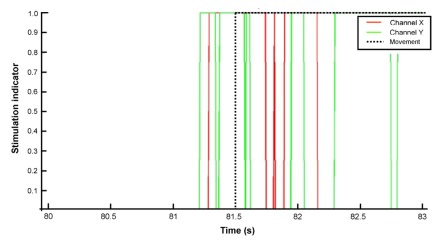
Prediction of the movement on the basis of the kinematic module (see also Figure 2). Red and green lines represent the predictions from the x-axis and y-axis, respectively. The black vertical dotted line corresponds to the voluntary movement. In this case, the movement is predicted approximately 250 ms in advance with the y-axis of the gyroscope (green).

**Figure 7.**
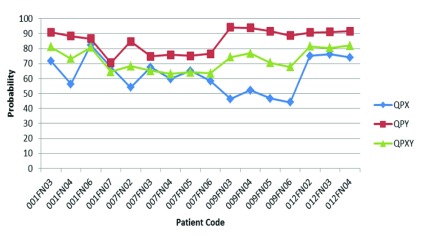
Kinematic QPs. Results for axis x, y and xy combined. Note the better results from channel y.

**Figure 8.**
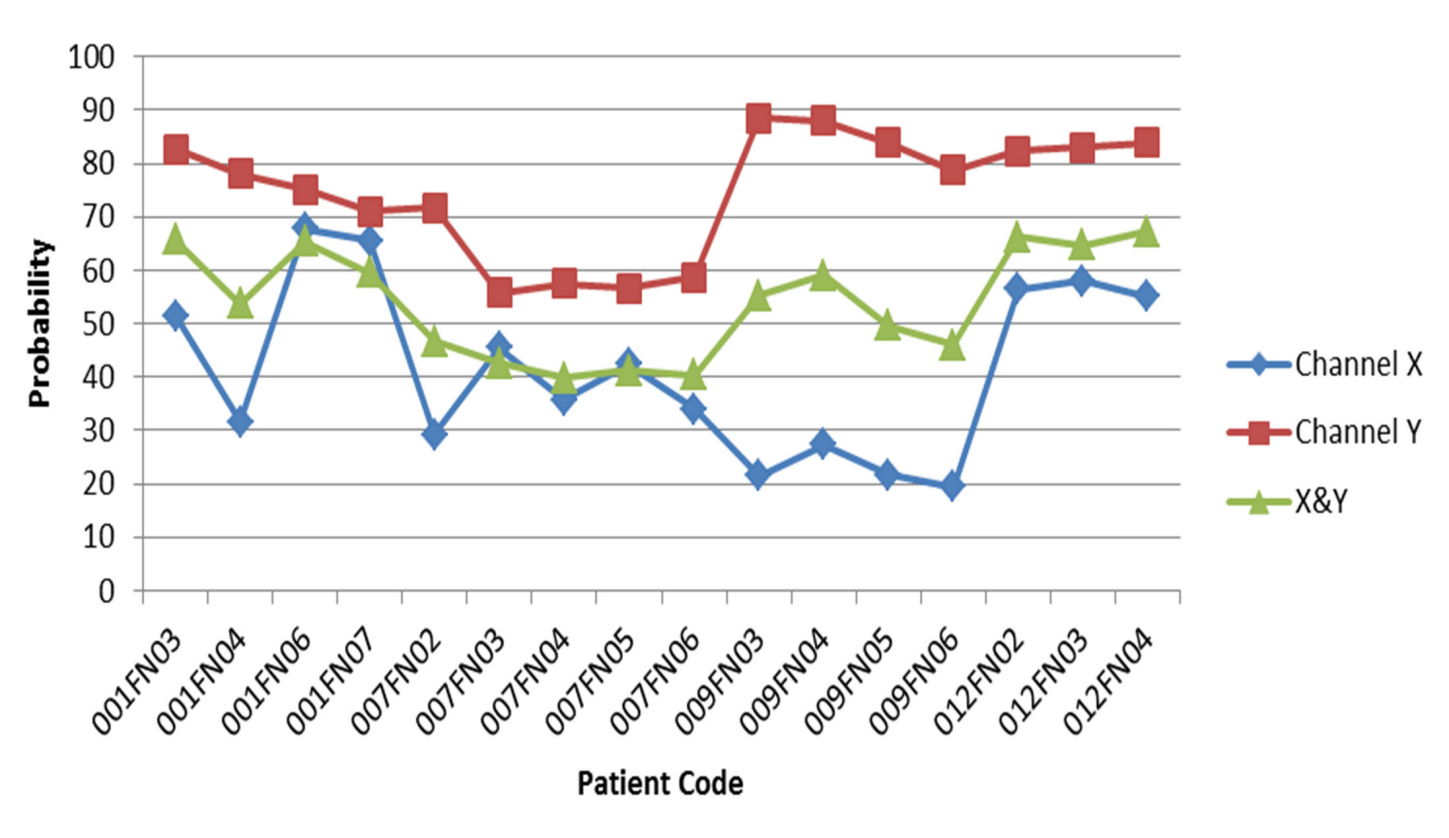
Probability of stimulation from the kinematic module for axis x, y and xy combined.

The selection of the axis of tremor is extremely important in this module. Indeed, if a patient has a pure mono-axial tremor on x-axis then better results are expected for this axis (as compared to the y- or z-axis). In our group of patients, the y-axis provided better results. Figure 6 shows the results of predictions of movements with the kinematic module. Figure 7 shows a comparison of kinematic QPs for the x-axis, the y-axis, and a combination. Values above 70% were reached for the y-axis. Figure 8 illustrates the probability of stimulation (see the *Kinematic module section in Materials and methods*). Clear differences between the x-axis and the y-axis were observed.

### D. Global multimodal plot

**Figure 9.**
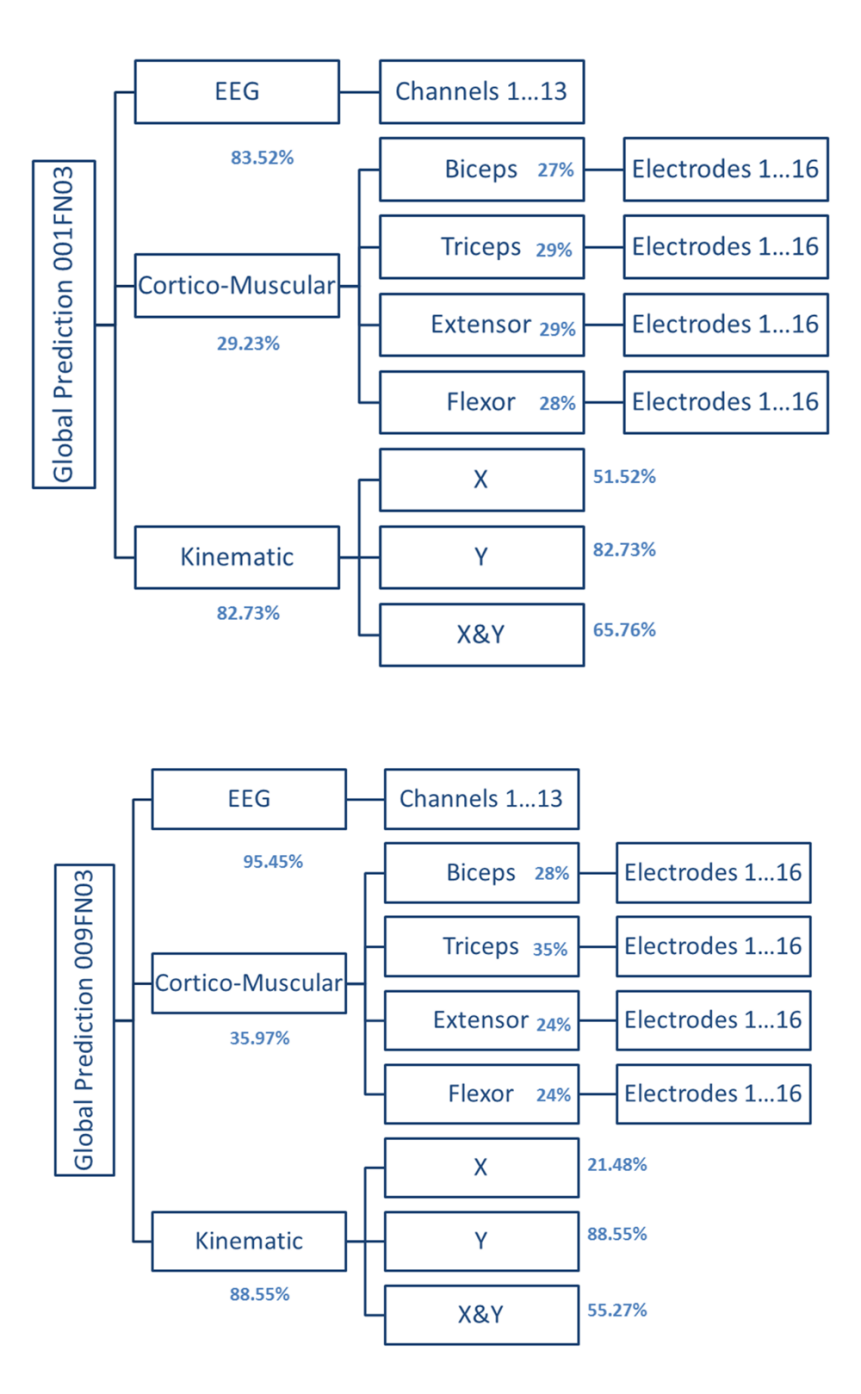
Example of probability tree from patients 001 and 009 (trials FN003). EEG QPs and the probabilities of stimulation for the kinematic and the cortico-muscular modules are visualized in a tree form. For each leaf of the tree, a parameter of prediction can be computed. Values are given in %. Once the tree is filled, it is possible to identify the best parameters and associate them.

To select the appropriate parameters for the BCI, a probability tree was built in order to identify the best associations of parameters. As an example, probability trees from 2 patients are shown in Figure 9. The probability can be extended with several combinations (probabilities for each EEG channel, each EMG electrode or combinations). From the statistical point of view, it is important to note that in some cases the association of several parameters can worsen the prediction of the intentionality of movement, as compared to a single parameter. For example, the association of the channel x and y in the kinematic module yielded lower statistics than the y-axis alone ( Figure 9).

### E. Simulation of ERD/ERS: which thresholds would be required to obtain high EEG QPs?

**Figure 10.**
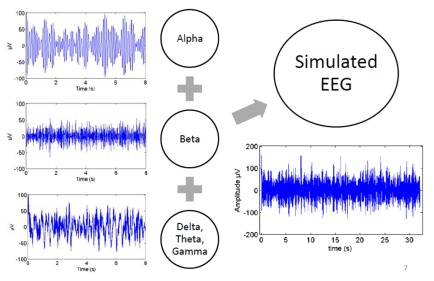
Method used to generate an artificial EEG for the simulation study.

**Figure 11.**
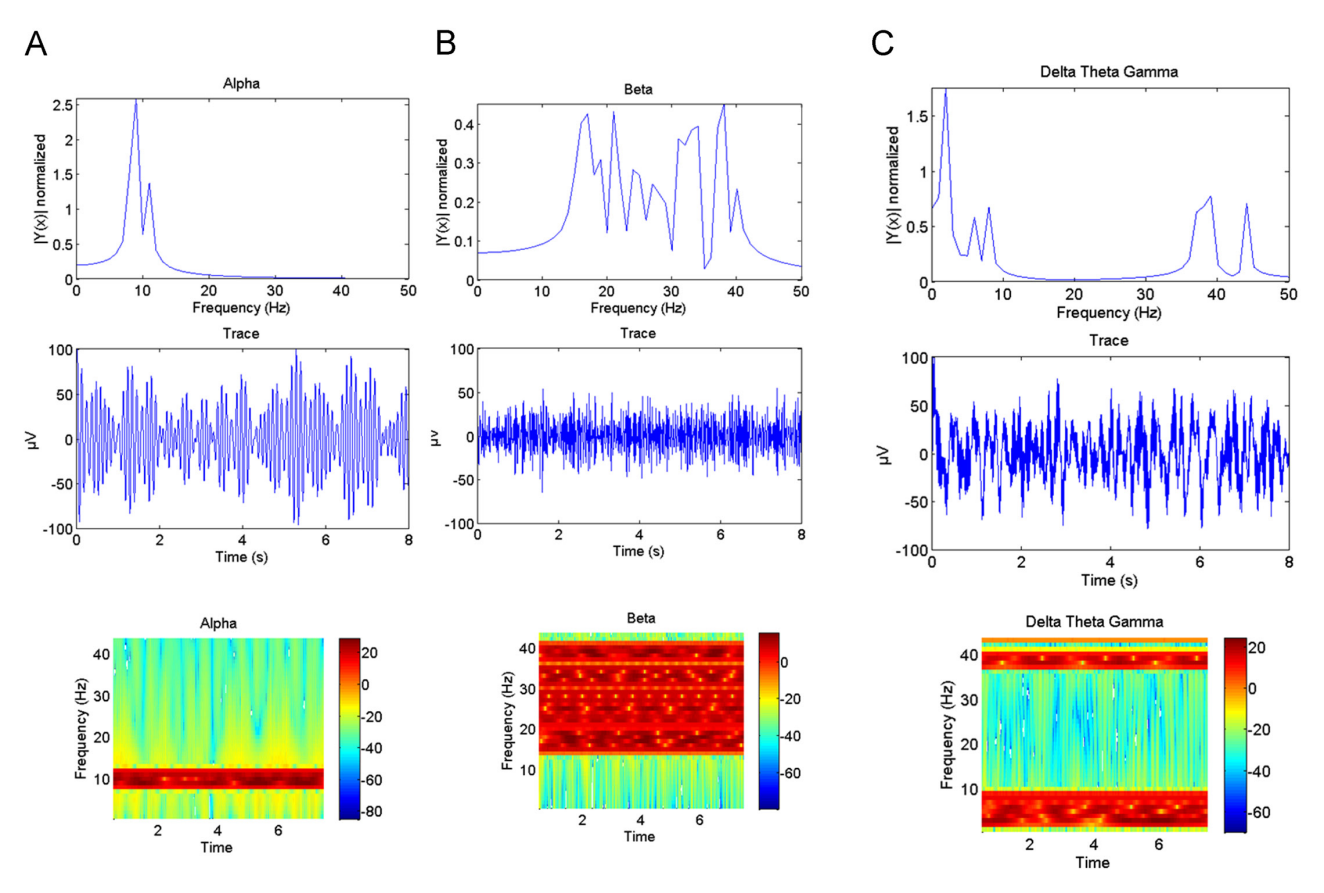
Example of spectral analysis of an artificial EEG containing alpha ( **A**), beta ( **B**) and delta theta gamma sub-bands ( **C**). A color-code is used for the representation of spectral densities (bottom panels). Note the red bands corresponding to the highest spectral densities.

**Figure 12.**
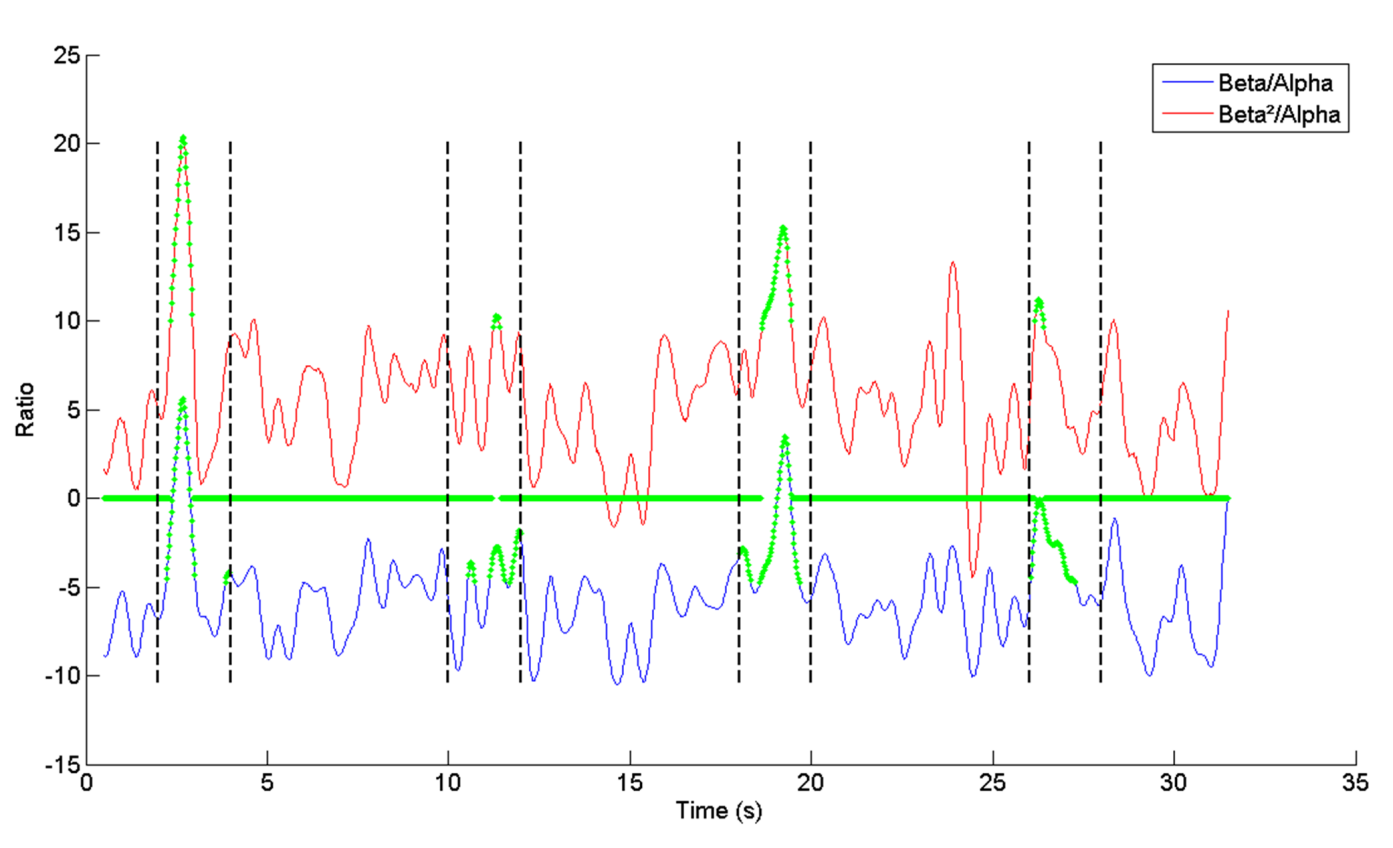
True positive values (green diamonds) for 4 events of desynchronization of the EEG (represented by vertical dotted lines). Blue trace: ratio beta/alpha; red trace: ratio beta²/alpha.

**Figure 13.**
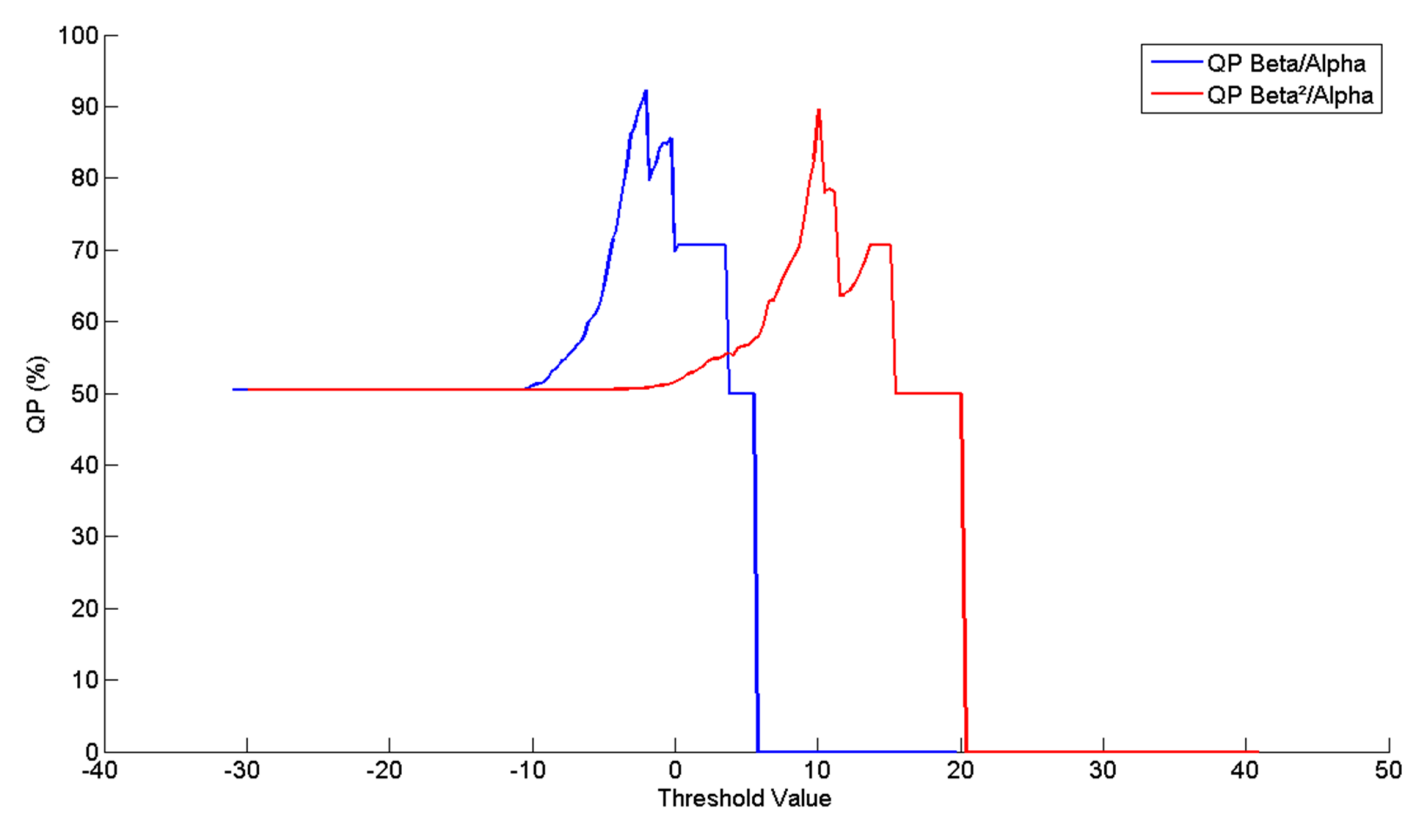
Relationship between the threshold of ERD/ERS and the QP. Threshold varying from mean – 10*SD to mean + 10*SD (steps of 0.1). QP is expressed in %.

**Figure 14.**
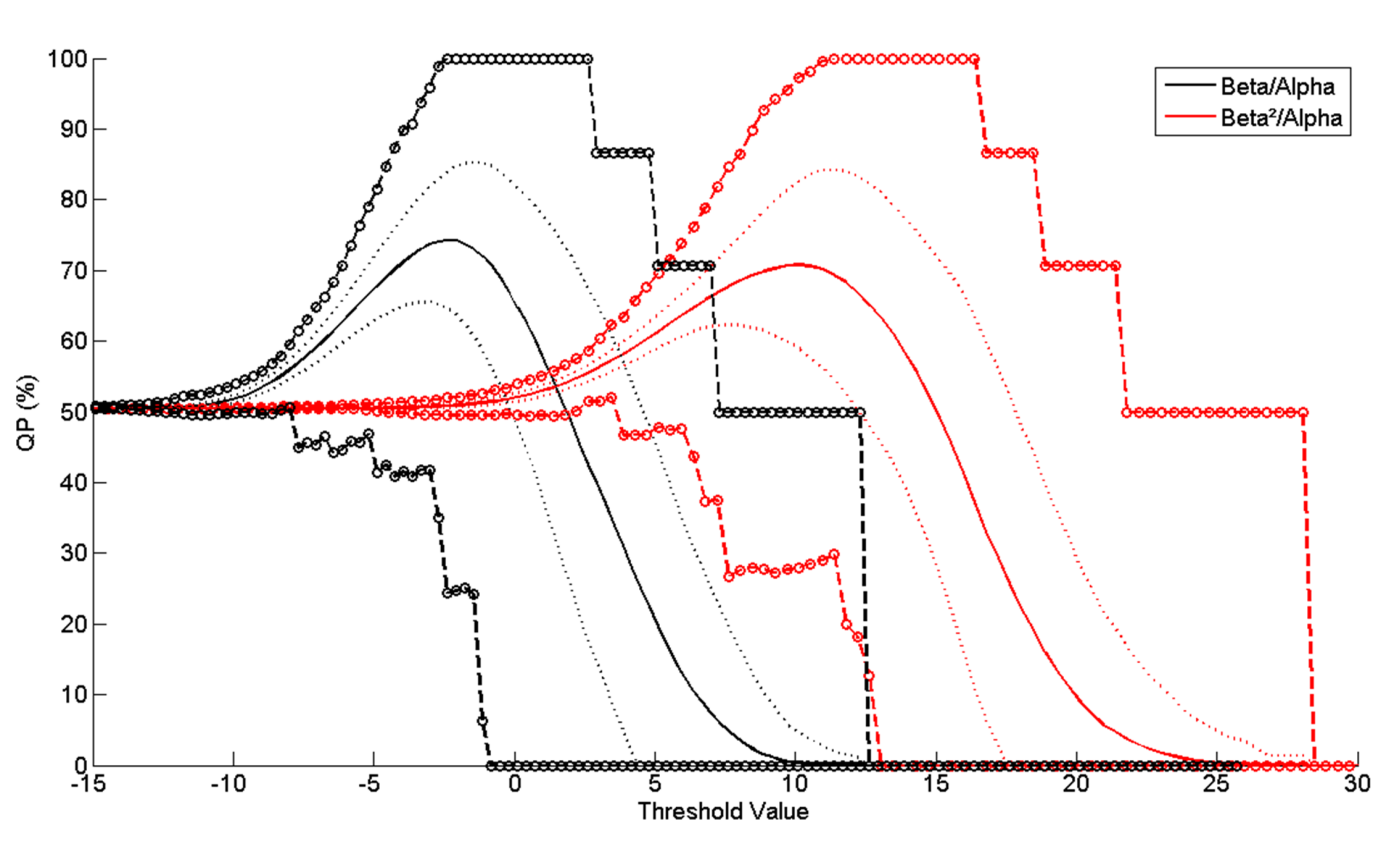
QP obtained as a function of the threshold of ERD/ERS used. Continuous trace: mean values. Dotted lines: mean ± SD. External lines with larger dots: 95% confidence interval.

One main issue and challenge for the use of a BCI-based on ERD/ERS in neurological patients is to predict whether a given patient would exhibit a sufficient ERD/ERS to be enrolled in therapies based on BCIs. To this aim, we simulated an EEG signal and specifically looked for the relationship between ERD/ERS and QPs. EEG signal was simulated according to a method reported earlier ^17^. The signal generated was a sum of four sinusoids with frequencies chosen randomly from specified ranges of frequencies (delta, theta, alpha, beta, gamma), with a random initial phase. The phase of the oscillations was reset at a specified timing for the simulation. The following parameters were used for generation of EEG signals: sampling frequency of 250 Hz, range of delta band: 0.5–4 Hz, range of theta band: 4–8 Hz, range of alpha band: 8–13 Hz, range of beta band: 13–40 Hz, range of gamma band: 36–44 Hz. Segments of 8 seconds were generated for each of these bands and were superimposed to obtain an artificial EEG trace ( Figure 10 and Figure 11). We repeated this procedure to obtain an EEG signal of 32 seconds. The spectrogram was computed with the Goertzel algorithm between 0.5 and 44 Hz (window of 256 samples, overlap of 250). A number of four events of desynchronization (with a duration of the desynchronization period of 2–4 sec for each of them) were introduced. Ratios beta/alpha and beta²/alpha were extracted. Figure 12 illustrates an example of the true positives (in green) for the QP for a threshold of desynchronization set as -4.8 and 9.5 for the simple and squared ratio, respectively. The threshold varied from mean – 10*SD to mean + 10*SD, using steps of 0.1. We performed a simulation for 2875 trials similar to the trial shown in Figure 12. Figure 13 illustrates how QP evolved as a function of the threshold values used. The traces of averaged QPs were characterized by values around 70% ( Figure 14). Very interestingly, these values are much greater than the values obtained for the corticomuscular module alone.

EEG, EMG, accelerometer and gyroscope data for a FES-mediated brain-computer interface (BCI) aiming to suppress neurological tremor 001FN03.mat_EEG.csv EEG data of patient 001FN03. 15 EEG channels (Fs: 1000Hz) : FC3, FCz, FC4, C5, C3, C1, CZ, C2, C4, C6, CP3, CPZ, and CP4 (POz: ground; linked ear-lobes: reference). Each column represents the above channels in the cited order.
001FN03.mat_IMU.acce.csv Accelerometer data of patient 001FN03. 3 channels (Fs: 1000Hz) : x, y, z. Those data are presented here as complementary information. Scroll down sheet to view values.
001FN03.mat_IMU.gyro.csv Gyroscope data of patient 001FN03. 3 channels (Fs: 1000Hz) : x, y, z. This is used for the kinematic module. The first second (more or less 1000 records) is silent and corresponds to the difference in time made while turning on the different acquisition devices. Scroll down sheet to view values.
001FN03.mat_sEMG.biceps.csv Biceps EMG data of patient 001FN03. 15 channels (Fs: 1000Hz) : Resampled EMG data. This is used in the cortico-muscular module. Crossed with EEG data, cortico-muscular coherence has been computed for all possible couples of EEG-EMG data.
001FN03.mat_sEMG.extensors.csv Extensors EMG data of patient 001FN03. 15 channels (Fs: 1000Hz) : Resampled EMG data. This is used in the cortico-muscular module. Crossed with EEG data, cortico-muscular coherence has been computed for all possible couples of EEG-EMG data.
001FN03.mat_sEMG.flexors.csv Flexors EMG data of patient 001FN03. 15 channels (Fs: 1000Hz) : Resampled EMG data. This is used in the cortico-muscular module. Crossed with EEG data, cortico-muscular coherence has been computed for all possible couples of EEG-EMG data.
001FN03.mat_sEMG.triceps.csv Triceps EMG data of patient 001FN03. 15 channels (Fs: 1000Hz) : Resampled EMG data. This is used in the cortico-muscular module. Crossed with EEG data, cortico-muscular coherence has been computed for all possible couples of EEG-EMG data.
QPs.csv Quality paramter vs threshold numerical values (simple ratio). This is to find exact values corresponding to what is observed on the (corresponding) figure of the article. Each column is a different threshold value.
QPsq.csv Quality paramters vs threshold numerical values (squared ratio). This is to find exact values corresponding to what is observed on the (corresponding) figure. Each column is a different threshold value.
Ts.csv Intermediate threshold results. This can be discarded.
Tsq.csv Intermediate threshold results. This can be discarded.

## Discussion

We present a novel method to predict the intentionality of movement in neurological patients presenting tremor in the upper limbs. We use a multimodal approach based on the combination of several parameters, in order to decrease the rate of false positive and false negative detections. Starting from the EEG and the kinematic signals, we have extracted a QP, defined as the geometric mean of the probability of movement prediction and the number of movements detected. For the EEG module, the extraction of QP is based on the changes in ratios of sub-bands according to the ERD/ERS phenomenon. We suggest that values equal or higher than 70% correspond to a good QP, as compared to values in the literature ^18, 19^. QP values greater than 90% were observed in some of the runs performed by our patients. However, an inter-patient and intra-patient variability was found and further evaluations with a larger number of patients and more runs per patient are required. The complexity of EEG recordings in patients with tremor performing upper limb movements should not be underestimated, especially when tremor genesis involves deep nuclei in the brain.

Our protocol in neurological patients with tremor differs from those in the literature, hence our study on the multiple combinations of frequency bands. When a neurological patient with tremor is seated and assessed, he/she may exhibit a tremor of the head and trunk. This tremor may be pretty stable or rather intermittent. There may even be an overlap with the main frequencies of the EEG signal, for instance in the alpha band (a rapid head tremor may be found in patients). Therefore, we decided to have a close look to each of these bands. For instance, we have seen patients with cerebellar disorders and orthostatic tremor in whom the sub-band 8–10 Hz was much less informative as compared with the sub-band 10–12 Hz. We would like to point out that in the study of Pfurtscheller *et al.* on single-trial classification of EEG and imagination ^20^, the frequency of the most reactive components was 11±0.4 Hz (mean±SD). The SD was thus small. Although the desynchronized components were centered at 10.9 Hz±0.9 Hz, the synchronized components were narrow-banded, with higher frequencies at 12.0 Hz±1.0 Hz. We agree with the authors that the classification of single EEG trials improves when ERD and ERS patterns are combined for multiple tasks. We aim to pursue the use of narrow bands of frequency in multiple tasks.

The QP parameter has been defined as a geometric mean in order to force both the true positive stimulation rate (in case of FES application) and the percentage of detected movements to be high enough to obtain a good QP value. Adaptive algorithms could be implemented to take into account variations of the standard deviation and, thus, to adapt to different kinds of activities that have different ratio profiles. We suggest that the choice of the thresholding method and the convenient sub-band ratio for the application of QP in the framework of a BCI-driven system should be made for each patient, depending on the neurological disorder considered. Neuroscience and engineering research support the hypothesis that the inclusion of non-invasive EEG data in the pre-movement period (which corresponds to motor preparation and planning) is useful to reach more effective rehabilitation procedures and to decrease the response time of BCIs ^21^. It is very likely that the design of more advanced neuroprostheses and robot-assisted neurorehabilitation will benefit from EEG-based BCIs ^22^. Techniques of multichannel EEG compression, phase congruency and graphical representations aiming at a reduction of multidimensional data have been proposed ^23– 25^. However, no technique has been widely accepted so far.

In theory, BCI is an interface between brain and computer. As such, our system would be a multimodal control unit, including an EEG-module like often used for BCIs, but also body modules to control a stimulation unit. Future works could apply some feature selection algorithm and train the multimodal control unit in discriminating movements based on the multimodal input. These two steps could further be included in one step e.g. by use of random forests. By doing so, the performance of the modules would be evaluated to find out which ones contribute most to a high detection rate. This would be done separately for each patient, thus taking into account the inter-individual variability.

The study of the kinematic data has revealed interesting features in terms of detection of voluntary movements in patients with rest tremor. This tremor occurs mainly in extra-pyramidal disorders such as Parkinson’s disease, which is a very common neurological disorder in the elderly. Assessments of kinematic data per se are particularly interesting because of their simplicity and their direct access without intrusion in the body. Three main neuronal mechanisms have been hypothesized for rest tremor: a cortico-subthalamo-pallido-thalamic loop generating tremor, a pacemaker consisting of the external pallidum and the subthalamic nucleus, and an abnormal synchronization within the whole striato-pallido-thalamic pathway leading to a loss of segregation ^26^. The findings of a decrement of rest tremor before voluntary movement in Parkinson’s disease patients suggest an involvement of a neuronal input from the cerebellum to the thalamus, which may occur sufficiently early to suppress the resting tremor before the voluntary movement ^11^. However, it remains unclear how the understanding of these oscillators in the brain will impact directly on the design of BCIs.

Probability trees show a global visualization of the parameters proposed for the prediction of movement and allow the identification of the best ones (or the best association of them). When all the possible combinations of EEG/EMG/kinematic QPs are tested, the probability trees could yield an optimal efficiency. An exhaustive list of the probabilities for the entire amount of data recorded is not provided, because of the huge amount of time that this analysis requires in terms of data processing. The global multimodal plot improves the effectiveness of the system by providing redundant parameters for the prediction of movements. Moreover, it could be particularly helpful during the training phases of the BCI implementation in a given patient. These training phases are known to be time-consuming in some patients. Our data provide a ground for the concept of multimodal approach developed for the early detection of the intentionality of movement. The presented probability trees are general schemes. A case-by-case analysis is required. In order to provide the most possible accurate BCI-driven FES system, each subject needs to be studied in order to define the best combination of QPs. For instance the kinematic QPs may be more efficient than the EEG QPs in a given patient (as it may happen when ERD/ERS is not stronger enough to be detected). The system would take into account these features. By analysing a larger group of patients, we might identify subgroups of patients on the basis of the results of the probability trees. In other words, the probability trees would be used as an elegibility procedure to multimodal BCI-driven treatments in neurological patients with tremor.

Results obtained with the simulation study provide useful information about EEG QP in order to select patients more effectively for a BCI-based treatment, including rehabilitation. The simulation demonstrates the relationship between the threshold and the QP. Future studies could take advantage of these findings to select the best neurological candidates on the basis of the ERD/ERS for BCI-based management.

**Figure 15.**
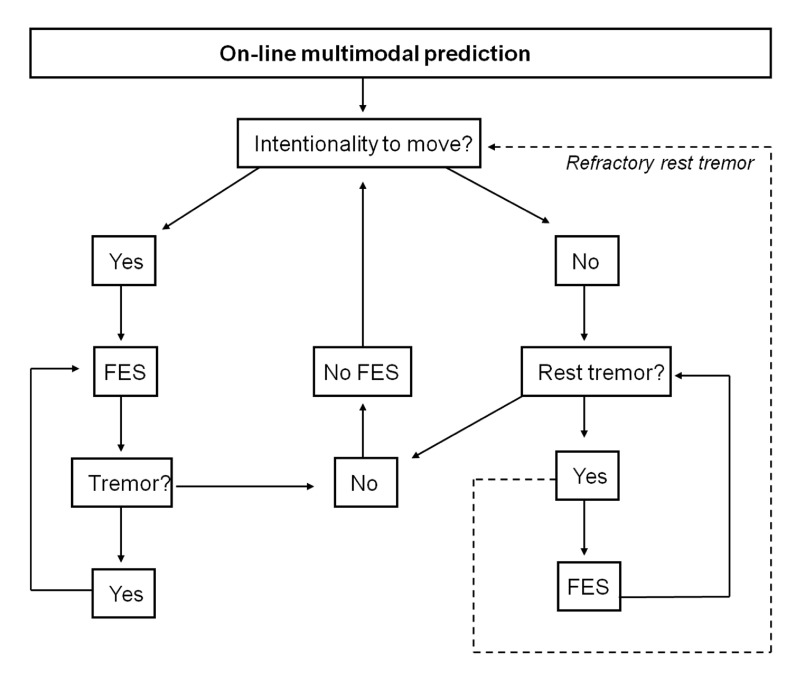
Proposal of a closed-loop approach for the detection of intentionality of movement and the triggering of FES. The detection of the intentionality to move is based on the quality parameters (QPs) reported here.

In patients responding to FES, we propose a novel closed-loop approach ( Figure 15). FES is applied to the upper limbs following the detection of the intentionality to move by the multimodal platform reported here and taking into account the analysis of the QPs, in order to prevent the emergence of tremor just before the start of action. FES is triggered to reduce or cancel tremor. In case of detection of rest tremor by the kinematic sensors, FES is applied accordingly to the muscles in the upper limbs. The parameters of FES (intensity of stimulation, duration of stimuli, modes selected) are adapted according to the severity of tremor and the tolerance. Refractory rest tremor may occur in patients in whom FES is not effective to suppress tremor. In these patients, the on-line multimodal prediction is used to trigger FES once the intentionality to move is detected. Indeed, the various forms of upper limb tremor may react differently to FES due to their distinct pathogenesis.

## Conclusion

We suggest a multimodal approach to identify the intentionality of movement. The QP is a promising index in the field of the ERD/ERS-based methods to detect the intention of movement for future BCI applications. This parameter could be also used to process EEG recordings from wearable dry electrodes. Novel wearable devices developed for the treatment of motor disturbances outside the field of neurological tremor might benefit from this approach. We propose that the EEG QP can be complemented by the QPs extracted from the cortico-muscular coherence and the QPs obtained by the analysis of the changes in the kinematic signals, which occur prior to the voluntary movements. We suggest a fusion of the QP parameters in order to increase the likelihood to detect the intentionality of movement. The analysis of the corticomuscular coherence shows that this parameter alone cannot be used to predict voluntary motion and be implemented in a BCI. Global multimodal plots may become attractive with the development of wearable technologies. They will have to take into account the various pathologies of the central nervous system, especially the localization of the lesions and their course with time. It is very likely that in progressive neurological disorders, the parameters selected in global multimodal plots will have to be modified or adapted accordingly. This is in agreement with adaptive methods which are being developed currently with the goal of improving the classification algorithms for BCI system in order to extract EEG patterns related to a cognitive or motor status ^6, 27^. Our approach will have to be tested in a large sample of patients in the future, in order to demonstrate its real clinical usefulness in daily practice. We propose to select a larger group of neurological patients to confirm the strength of the multimodal prediction. The present study opens the door for future studies in terms of how to increase EEG-based detection of movement intention by incorporating information from multiple modules.

## Data availability

*figshare*: EEG, EMG, accelerometer and gyroscope data for a FES-mediated brain-computer interface (BCI) aiming to suppress neurological tremor, http://dx.doi.org/10.6084/m9.figshare.879661 ^28^
