# Supplementary material for: Quality parameters for a multimodal EEG/EMG/kinematic brain-computer interface (BCI) aiming to suppress neurological tremor in upper limbs
Source: F1000Res. 2014 Apr 30;2:282. Originally published 2013 Dec 20. [Version 2] doi: 10.12688/f1000research.2-282.v2 (PMC4193395; doi:10.12688/f1000research.2-282.v2)
Supplement: Peer review history

# Peer review: Referee response for version 2

**DOI:** 10.5256/f1000research.4274.r5149

**Published:** 2014-06-17

**Authors:** Carlos M. Gomez

**Affiliations:** 1 Human Psychobiology Lab, Experimental Psychology Department, University of Seville, Sevilla, Spain

The paper has clearly improved in clarity. As there are no statistics or the determination of an algorithm to predict the intentionality of movement, it is still a step to obtain the goal of controlling tremor by electrical stimulation.

The less clear part of the manuscript is the EEG simulation, if they do not have a neuro-muscular model of the tremor and its suppression by beta/alpha ratios is not clear how it is possible to estimate a false positive in the movement prediction. Also the EEG model is a descriptive, not a neurophysiological model.

With these reserves and positively scoring the obtained goals by the authors, it is an interesting contribution to find an on-line algorithm for predicting intentional movements in Parkinson diseases.

I have read this submission. I believe that I have an appropriate level of expertise to confirm that it is of an acceptable scientific standard.

# Peer review: Referee response for version 2

**DOI:** 10.5256/f1000research.4274.r4610

**Published:** 2014-05-07

**Authors:** Yvonne Hoeller

**Affiliations:** 1 Christian-Doppler-Clinic, Paracelsus Medical University, Salzburg, Austria

Again, I have to emphasise that this is interesting research and I found the detailed answers to my concerns very useful. I assume there were some minor misunderstandings in this large revision and some major concerns remain.

1. I suggested including frontal electrodes because we recently found that they are indeed important for detecting motor imagery. I agree with you that preprocessing is needed when using these electrodes. I would at least use frontal electrodes in your future work, even if you decide not to include them into your analysis (which I would really regret because I think the results would benefit from it).
2. I have a concern about studies in which the participants are told not to blink during a specified period. Participants are additionally engaged to concentrate on not blinking. Since motor imagery is a demanding task this could cause some bias. I would mention this in the limitations section.
3. I asked you about the up-sampling because I found that the explanation in the paper " *Upsampled EEG data were processed with a Hamming window of 256 samples, using an overlap of 250 in the time domain*" with the subsequent mentioning of 1000 Hz sampling frequency in some following paragraph is a little confusing. The intention of my questions was to guide you to the details of the paper which are unclear. By changing the text according to my questions, the paper could become more understandable. In other words: I did not only expect an answer to this question, I expected some changes in the text. This applies to all the other questions I raised in the first review. I would kindly ask you to change the manuscript according to my questions raised upon the first version. If readers can quickly understand what you have done, they are more likely to cite your work.
4. You wrote in your answers: " *We represent here the QPs that reached a good value of accuracy*." It is recommendable to state this in the paper together with a quantitative criterion for a good value of accuracy.
5. In general I find it difficult to see the statistics which were performed. In the results you state that there is " *no* *significant difference between the QP calculated from β/α ratio and β²/α ratio*" but there is no information on what test was used and which electrodes were tested etc.
6. For evaluating accuracy, you should use statistics instead of "good" accuracy above 70%, which comes out of the blue and may not be sufficient. There is a statistical test to assess if accuracy is significantly above chance. You could use the maximum chance criteria and an adequate measure of significance and effect size, as described in Marcoulides G, Hershberger S (1974) Statistical *Methods: A first course*. Psychology Press.
7. With respect to Figure 3 you answered that these data correspond to the central area of the brain. Is this data of a single electrode? You should exactly indicate this information in the revision.
8. Although you discuss the issue of BCI and multimodal interfacing, I find it still misleading to just read BCI in the title/abstract. This is simply the wrong term. You could change the title: Quality parameters for a multimodal human-computer-interface based on EEG, EMG, and kinematics, aiming to suppress neurological tremor in upper limbs.
9. Although you justify the selection of 2 patients and 3 trials, it needs to be discussed that this may not be representative. Thus, generalisations are limited. I would emphasise the exploratory character of this study in the title or at least in the abstract and the discussion.

I have read this submission. I believe that I have an appropriate level of expertise to confirm that it is of an acceptable scientific standard, however I have significant reservations, as outlined above.

# Peer review: Referee response for version 1

**DOI:** 10.5256/f1000research.3288.r3358

**Published:** 2014-01-27

**Authors:** Carlos M. Gomez

**Affiliations:** 1 Human Psychobiology Lab, Experimental Psychology Department, University of Seville, Sevilla, Spain

This report tries to identify EEG, EMG and kinematics features that permit prediction of movement production, in order to use functional electrical stimulation (FES) to compensate for tremor in neurological patients. The rational and the methodology is sound, but several points must be clarified in order to improve the validity of the result and to increase readability of the results of these important and worthy results, not only for academics but also in the clinical settings. The queries can be answered, clarified or considered for more advanced reports in the future.

**Introduction:**

- In Figure 1 the dashed line seemed to indicate the movement, but how it is possible that the movement itself is predicted by the movement kinematic - that should be zero before movement. Except if some thresholds are defined, pre-thresholds movements are the predictors. Please clarify the sentence "decrement of the rest tremor before movement onset" - does it means that the reduction in tremor is not the initial voluntary movement but a different neural command which is being inhibited by the voluntary control? Then define physiological characteristics, frequency amplitude, of tremor. Otherwise it would be difficult to distinguish between the reduction of tremor and the initial phases of movement.

**Methods:**

 

- "Mean age of the patients was 62 ± 20 years" . It would be more precise to describe the age of each individual subjects. Means are useful for high number of subjects. Same for other parameters.
- "After (1) hearing an acoustic signal, the patient (2) prepared themselves mentally for the execution of movement and (3) performed the task." It is not clear how long it takes this preparation. Was it induced by the experimenter instructions or it was an spontaneous strategy? How long does each of the phases take?
- Is it neccesary to indicate the files code? Otherwise please suppress it.
- "EEG signals were sampled at 256 Hz (re-sampling at 1000 Hz for synchronization purposes). " It seems that the data were re-sampled to a higher frequency. Could it be considered an interpolation rather than a re-sampling? Anyway it would be better to sample at 1000 Hz, if needed.
- Figure 2 is very difficult to follow, maybe doing a composite figure with accelerometer, gyroscopes and magnetometers separated would be better. One example of tremor suppression would be appreciated.
- "The peaks (β/α and β²/α ratios) higher than a defined threshold were considered as indicators  of a potential voluntary movement.  given that they represent the detection of the cortical motor preparation of the movement" The authors are very confident with this option, but some information should be given to the non-specialist of BCI.
- "Three time intervals were studied: pre-movement period, movement period, post-movement period. The pre-movement period (lasting 2 seconds) was defined according to the acoustic order given to the patients and the detection of the beginning of movement via the gyroscopes." Please give a more precise description of the time window analyzed - 2000 ms before the movement? 2000 ms after the auditory signal? In the middle? Was there always 2000 ms between auditory signal and movement?
- "The peaks (β/α and β²/α ratios) higher than a defined threshold were considered as indicators of a potential voluntary movement" Was the threshold pre-defined *a priori* (which value?), or adjusted following the predictive value.
- "More than 800 possible EEG/EMG combinations (n=832)", please clarify the origin of these 832 combinations.
- "Where *p *is the probability of movement and *n *the number of movements predicted", please define more precisely the probability of movement.
- In general, it would be desirable to have an experimental protocol in which the subjects have the opportunity to decide if he/she wants to move or not. Or still better to go to a more ecological situation in which the subject is instructed to do the finger-to-nose movement at its own pace. And compare false positive and false negative predictions.

 

**Results:**

 

- "The mean QP was 82±12% (median = 83.5%) for the β/α ratio and 79.5±10.4% (median = 80%) for the β²/α ratio." Please report individual subjects' values.
- Figure 3 reports values of QP much lower than the mean values reported in the text. Please clarify.
- Which are the coherence values for Figure 4? The Y axis is missing.
- Figure 6: Is not the predicted predicting? Or, is not the movement pre-threshold predicting the post-threshold movement? Is that the reason why the Y channel is so good predictor of movement? Please clarify. If it is reduction of tremor, please show some examples.
- The motivation of the simulation is not clear, because only the EEG can be simulated, not the movement. Please clarify the interest of the simulations study or remove it.

 

**Discussion:**

The discussion seems to clarify that the excellent predictor of reduction of tremor is produced by a different command than the voluntary control. But tremor should have a definite frequency, and this frequency property of the tremor seems not be taken in account in present report.

I have read this submission. I believe that I have an appropriate level of expertise to confirm that it is of an acceptable scientific standard, however I have significant reservations, as outlined above.

# Response

**Published:** 2014-04-24

**Authors:** Mario Manto

**Affiliations:** FNRS-Laboratoire de Neurologie Expérimentale, Hôpital Erasme, Université Libre de Bruxelles, Belgium

We thank the referee for their criticisms.

**Introduction:**

- *“In Figure 1 the dashed line seemed to indicate the movement, but how it is possible that the movement itself is predicted by the movement kinematic - that should be zero before movement. Except if some thresholds are defined, pre-thresholds movements are the predictors. Please clarify the sentence "decrement of the rest tremor before movement onset" - does it means that the reduction in tremor is not the initial voluntary movement but a different neural command which is being inhibited by the voluntary control? Then define physiological characteristics, frequency amplitude, of tremor. Otherwise it would be difficult to distinguish between the reduction of tremor and the initial phases of movement.”*
  The initiation phase of movement in a patient with rest tremor has been characterized in details in the literature (Papengut *et al. *2013). In patients exhibiting a rest tremor, tremor suppression at movement onset (reduction in amplitude or complete cessation, detectable with kinematic sensors) is an example of noticeable changes in tremor features occurring at the beginning of the voluntary movement: tremor features change and this is detectable by kinematic analysis. It is presumed that the voluntary command inhibits the central oscillator generating rest tremor. Since we are using both accelerometry and gyroscopes, the beginning of the finger-to-nose movement is clearly identified by changes in kinematics.
  The introduction and the legend of Figure 1 have been changed. 

**Methods:**

- *“"Mean age of the patients was 62 ± 20 years". It would be more precise to describe the age of each individual subjects. Means are useful for high number of subjects. Same for other parameters.*
  We have included a table detailing age and other parameters in the Methods of the revised article. Patients were followed on a regular basis during this European project. The clinical scores were pretty stable, although slight clinical fluctuations were observed (this is very common in the follow-up of neurological patients exhibiting a tremor in the upper limb).
- *“"After (1) hearing an acoustic signal, the patient (2) prepared themselves mentally for the execution of movement and (3) performed the task." It is not clear how long it takes this preparation. Was it induced by the experimenter instructions or it was a spontaneous strategy? How long does each of the phases take?”*
  The text has been changed to the following:
*“Patients were told to keep the most relaxed attitude. After hearing an acoustic signal, they prepared themselves for the execution of movement by mental imagery of the movement. During a single run, the task was repeated about 10 times.” *
  The preparation phase lasted typically between 2 and 8 sec. There was variability between trials depending on self-estimation by patients. This is typical in mental imagery. Some patients are “fast-imagers” and others are “slow-imagers”.
- *“Is it necessary to indicate the files code? Otherwise please suppress it.”*
  This is useful in making the figures more readable (see Figures 7, 9 and 10) *.*
-  
*“* *"EEG signals were sampled at 256 Hz (re-sampling at 1000 Hz for synchronization purposes)." It seems that the data were re-sampled to a higher frequency. Could it be considered an interpolation rather than a re-sampling? Anyway it would be better to sample at 1000 Hz, if needed.*”
  Yes, we could consider an interpolation and/or a re-sampling. The rationale for the re-sampling at 1000 Hz is that it does not affect the spectral contents in the frequency sub-bands that are particularly of interest in our study. An interpolation technique is an alternative.
- *“Figure 2 is very difficult to follow, maybe doing a composite figure with accelerometer, gyroscopes and magnetometers separated would be better. One example of tremor suppression would be appreciated.”*
  We agree with the referee that this figure might be confusing. We have modified the figure in the revised article. The description of the sensors is reported in Gallego *et al.* (2010)
*S*ensors are a combination of triaxial accelerometers, gyroscopes, and magnetometers. The low weight of IMUs makes them an optimal solution, as tremor changes its characteristics if a larger mass is attached to the limbs. Moreover, their small size does not interfere with user’s movements.
  The phenomenon of tremor suppression induced by the application of FES has been published earlier. Here is an example of essential tremor responding strongly to FES. This patient does not respond to any medication (including: propranolol, primidone, topiramate). Although refractory to conventional drugs, this patient was FES-sensitive. 
  Figure from:
  Grimaldi G, Manto M: “Old” and Emerging Therapies of Human Tremor. Clinical Medicine Insights: Therapeutics. 2010; **2**: 169-178. Publisher Full Text. Published under the Creative Commons CC-BY-NC 3.0 license.
- *“"The peaks (β/α and β²/α ratios) higher than a defined threshold were considered as indicators of a potential voluntary movement given that they represent the detection of the cortical motor preparation of the movement" The authors are very confident with this option, but some information should be given to the non-specialist of BCI.”*
  This method is now explained in the revised text. The following reference has been added: Pfurtscheller and Lopes da Silva (1999). This methodology is considered as a sound procedure for the detection of the preparation of movement.
***“***"Three time intervals were studied: pre-movement period, movement period, post-movement period. The pre-movement period (lasting 2 seconds) was defined according to the acoustic order given to the patients and the detection of the beginning of movement via the gyroscopes." Please give a more precise description of the time window analyzed - 2000 ms before the movement? 2000 ms after the auditory signal? In the middle? Was there always 2000 ms between auditory signal and movement?** ***”***
  The following sentence has been added: ****“by considering 2 seconds back from the point of detection of the beginning of movement. We decided to use a period of 2000 msec based on the available literature which considers that 2 seconds encompasses the preparation phase at the cortical level.”**
- ***“**"The peaks (β/α and β²/α ratios) higher than a defined threshold were considered as indicators of a potential voluntary movement" Was the threshold pre-defined a priori (which value?), or adjusted following the predictive value.* ***”***
  This has been studied earlier. The best thresholding process should be decided in a case-by-case scenario, as described in Giuliana, Mario and Yassin (2011); % of maximum ratio overtime; threshold considering mean and standard deviation of the ratios.
- *“"More than 800 possible EEG/EMG combinations (n=832)", please clarify the origin of these 832 combinations."*
  The number of combinations corresponds to 13 EEG channels (excluding the 2 channels for the eyes) and 64 EMG channels (16 EMG channels for each of the 4 muscles of the upper limb)
  13x64 = 832 combinations
- *“"Where p is the probability of movement and n the number of movements predicted", please define more precisely the probability of movement.”*
  The text has been changed to the following:
*“Where p is the probability of movement (true positives) and n the number of movements predicted, as previously described for the EEG QP.”*
- **“In general, it would be desirable to have an experimental protocol in which the subjects have the opportunity to decide if he/she wants to move or not. Or still better to go to a more ecological situation in which the subject is instructed to do the finger-to-nose movement at its own pace. And compare false positive and false negative predictions.”**
  Thanks to the acoustic signal, the period of preparation of movement followed by the execution of the task is clearly defined. The patient knows that after the signal (1) he/she has to relax, (2) he/she has to prepare for  the movement, and (3) he/she has to execute it. It is important to note that it is much easier for neurological patients to follow this sequence with an acoustic signal and not to include an additional internal evaluation to move or not to move. The introduction of an additional decision to move or not is a source of complexity, which by itself interferes with the severity of tremor. Some neurological patients have difficulties and show hesitations for self-paced movements. The task is easier with the acoustic cue. See also the reply to Yvonne Hoeller.

**Results:**

- *“"The mean QP was 82±12% (median = 83.5%) for the β/α ratio and 79.5±10.4% (median = 80%) for the β²/α ratio." Please report individual subjects' values."*
This table shows the individual values.
- *“Figure 3 reports values of QP much lower than the mean values reported in the text. Please clarify.”*
  Figure 3 illustrates the effects of the selection of the sub-band beta upon the quality parameter. The figure illustrates values for one patient.
    
- *“Which are the coherence values for Figure 4? The Y axis is missing.”*
  The computed coherograms are shown using arbitrary units.
- *“Figure 6: Is not the predicted predicting? Or, is not the movement pre-threshold predicting the post-threshold movement? Is that the reason why the Y channel is so good predictor of movement? Please clarify. If it is reduction of tremor, please show some examples.”*
  There is very often one channel of the sensor which gives a much better predicting value. This rule can be applied to movement in general and to tremor in particular: one direction is much more informative (this is called the dominant axis). This explains why some groups consider that a single-axis sensor may provide relevant information *.*
- *“The motivation of the simulation is not clear, because only the EEG can be simulated, not the movement. Please clarify the interest of the simulations study or remove it.”*
  The simulation study is very important in order to have information about the strength of the ERS/ERD phenomenon. This parameter is important to state that a given patient having a strong enough ERS/ERD may be enrolled for a BCI-based therapy. This is a critical step, because some patients will benefit from a BCI-based management, whereas others not. We want to underline that we anticipate that in the near future this methodology will help to select patients for BCI programs. For the moment, some patients are enrolled and perform hours of training without success. An ERS/ERD-based decision to include patients in training programs will render the BCI-based management much more efficient. 

**Discussion:**

- *“The discussion seems to clarify that the excellent predictor of reduction of tremor is produced by a different command than the voluntary control. But tremor should have a definite frequency, and this frequency property of the tremor seems not be taken in account in present report.”*
  Tremor parameters have not been taken into account in this paper which is focused on the detection of voluntary movement. The overall concept of this European study was to predict the intentionality of movement and not to track the tremor parameters. FES is supposed to be active on the various forms of tremor. The point raised by the reviewer was addressed in detail in a previous European study (Manto *et al., *2003). Tremor is a very dynamic process, but the major advantage of FES is to have a non-selective suppressive effect by acting on the peripheral nervous system.

# Peer review: Referee response for version 1

**DOI:** 10.5256/f1000research.3288.r2883

**Published:** 2014-01-13

**Authors:** Yvonne Hoeller

**Affiliations:** 1 Christian-Doppler-Clinic, Paracelsus Medical University, Salzburg, Austria

This article presents a highly promising idea of using a multimodal control unit for FES to treat tremor during movement. I think this is a valuable contribution to the literature since the possible application (closed-loop FES) is an auspicious field of treatment research.

The manuscript is well-written and technically sound. However, due to lack of clarity I have several questions regarding the methods and the results. Moreover, given the exhaustive analysis of EEG frequency ratios and EEG-MEG coherence, I would suggest applying some feature selection methods in order to overcome the problem of multidimensional data, which is hard to present and to interpret.

**Abstract**

The sentence " *A BCI-driven detection of voluntary movement is used to trigger FES in a closed-loop approach.*" sounds to me like you have used FES in this study. Perhaps it would be less misleading if you wrote this in the subjunctive.

**Introduction**

In the introduction you emphasize the limitations of BCIs, which are based on conventional EEG analysis. Although I fully agree with you, this is a rather unspecific statement. Does this mean that "conventional" EEG analysis is less reliable for BCI-performance than "high-tech" EEG-analysis? Although we both know that this is not the case (BCIs are fed with highly sophisticated markers of the EEG and still do not perform at 100%), the sentence could be understood in this way. Therefore, I would suggest that you specify that you mean BCIs which ware based on EEG analysis only.

I would like to make a further point on this: BCI users differ between each other in the way they can voluntarily modify their brain activity, and there is even an interindividual difference in the detectability of movement-related EEG-activity. Moreover, the fact that movement disorders often show up with brain atrophy or neuroplastic changes makes it difficult to generalize BCI markers that have been tested in a healthy population to patients with varying pathologies. I think this variance is a major reason why your multimodal processing could do so much better than a BCI-system that is solely based on EEG analysis.

**Methods**

(A) I don't think that the sentence " *Patients and the experimental procedure were as detailed in the next sections.*" in the first paragraph of the Methods section is necessary (the reader sees that there is a section for patients etc.)
(A) Maybe in this first paragraph it would be nice to read that the different modules act during different time-windows.
(B) Why did you prefer acoustically triggered movement over self-paced movement? How did you ensure that the acoustic signal did not influence the EEG signal which was used for detection of movement planning?
(B) Why did you restrict your EEG-setup to centrally positioned electrodes? Movement planning involves frontal regions (e.g. electrodes F3, F4, F7, F8) and it is highly likely that you get a better prediction of movement if you include signals from these positions.
(B) How did you avoid blinking during the recordings? If you tell the participants that they should not blink, they might concentrate on this instruction instead on the task, making EEG-data less valid. In addition, participants who are told not to blink usually blink more frequently than if you would not mention blinks.
(Fig. 2) I did not understand the reason why you only used three colors for the lines in figure 2. The reader can distinguish the three cases but not which sensor is represented by what line. Moreover, the first blue line, entitled "Mouvement..." should be entitled Movement... and should be a dashed line as indicated below the figure. Are the three cases three different sensors, three patients or three movements?
(D) You write " *upsampled EEG data..*." and indicate that there were 256 samples - is this one second? Does an overlap of 250 in the time domain refer to the original sampling rate or to the upsampled data, resulting in 250ms? Did I get this right; the Hamming window was 1 sec but overlapped with 250ms?
(D) I do not exactly understand how you realized that the pre-movement period lasted for 2 sec, but participants performed the task following the acoustic signal. Were participants instructed to wait 2 sec? Or is this the average time the participants took until they performed the task?
(D) I did not understand what you intended by the (8 Hz, 9 Hz, ... 12 Hz) after the first interval.
(E) Possibly because this is the first time I have read about this very interesting method of correlating brain and muscle signals - I did not fully understand the segmentation/processing of the data. You segmented data into epochs of 200ms and calculated the FFT on this window - what then does the " *window of 8 samples*" mean? Moreover, how did you determine the optimal window length? I see that the epochs vary between the modules.

**Results**

(A) Why do you refer to reference no. 8 when you report your results? Aren't these your present results?
(A) The central areas showed the highest values of QPs - indeed, you used only a central montage?
(A) You talk about means - obviously, you may indicate averages over patients, but did you also average over electrodes? 
(Fig. 3) What electrode positions are used to build this figure?
(Fig. 4) The vertical dashed lines seem to occur periodically (each 20ms) with a certain time-distance but not necessarily on the highest peaks - do you have an interpretation/explanation for this phenomenon?
(Fig. 5) With what rationale were the 2 patients and 3 trials chosen?
(Fig. 6) It would be helpful to rescale the x-axis, so that the beginning of movement is time 0 and then indicate steps of +-250ms.
(Fig. 9) What is the EEG/CorticoMuscular probability based on - averages across channels/channel combinations or one single channel/combination (the best one?)?
(Fig 14) There are not only continuous and dotted lines but also external lines with "big dots" - is this the range (min-max)?

**Supplementary data**

What do the rows in e.g. 001FN03.mat_EE...csv mean? Are these samples?

**Discussion**

1. You state that QP values greater than 90% were observed in some of the runs...That is, QP was calculated for each run? This is not clear from the methods section.
2. Why are EEG recordings more complex when tremor is generated in nuclei deep in the brain?
3. In the discussion of kinematic data it seems to me that you discuss the background generally without referring to your own results. You should rather discuss why the y axis alone is superior to x axis or a combination of the two.
4. Do probability trees really show the best association of parameters? In a subsequent sentence you write that all possible combinations need to be tested. To me probability trees as used in this paper seem to give the impression of how complex the problem is, but they do not answer the question: Which feature/combination of features leads to highest detection rates? This problem is solved by feature selection algorithms (see general remark 2).
5. I am not an FES-expert, but wonder if there is any literature about the instance of time when FES has to be applied in order to make a movement free of tremor. Should it be applied at movement onset, or before the movement is carried out, i.e., in the planning phase? It probably depends on each individual patient, since tremor can occur pre- movement or during movement. Thus, the kind of FES should be different for each patient and similarly, depending on the type of application, the EEG-part may or may not be useful. Also if planning the action induced the tremor, detection of movement intention based on EEG could be too late?
6. There is no discussion about the simulation results.

**Conclusion**

1. In the conclusion it seems that the QP is an EEG-specific value which can be complemented by values from other modules (kinematic...) - instead, the QP is a value for estimating the performance in detecting movement and can be applied to each modality. But is this really a main conclusion? I think the conclusion should be that the EEG can be supplemented by other modalities. However, the extent to which the detection rate of the EEG can be supplemented, i.e., how much the QP can be increased by combining several modalities, has not been evaluated.
2. The last minus one sentence: The term classifiers comes out of the blue. This technique deserves some place in the discussion (as suggested in general remark 2).
3. Last sentence: The approach has firstly to be implemented - the present manuscript is a pre-study. The next step is to integrate all of the modalities. Then you could evaluate the approach on a large sample.

**General remarks**

1. You use BCI in terms of a control unit. BCI is an interface between brain and computer. As such, your system would be a multimodal control unit, including also an EEG-module like often used for BCIs, but also body modules to control a stimulation unit. I think the term BCI is not the best choice here, since it does not take into consideration the EMG module etc. 
2. The way such a problem would be solved by the current BCI-community is a classification by use of machine learning techniques i.e., you apply some feature selection algorithm and train your machine in discriminating movements based on the multimodal input. These two steps could further be included in one step e.g. by use of random forests. By doing so you evaluate the performance of your modules and find out which ones contribute most to a high detection rate - separately for each patient (thus, you take into account interindividual variability). I would at least include this option in the discussion.
3. I think the data presented here could help to determine what detection rate is possible by combining several modalities. This is what the reader could expect when they read the abstract and the introduction. Then, the conclusion could be that combining several modalities increases the detection rate or it does not change it or it decreases the detection rate (I expect that it increases it). Instead, the authors presented the detection rate by calculating a detection-rate affine measure (QP) separately for each module, and in the conclusion the authors just suggest to fusion the parameters. I think a deeper analysis (e.g. by using random forests...) could answer the very important question of how to increase EEG-based detection of movement intention by including information from other modules. I see that the authors want to leave this important question for future publications, but the abstract and the introduction should be clear in determining the aim of this study. The authors separately evaluated the value of multimodal parameters, in order to determine if it could be interesting to integrate them in one system.
4. The presentation of the results is rather descriptive, the authors report QP, SDs etc. but no statistical test is applied. It would have been interesting if the QPs differ between modalities, within modalities with respect to electrode locations etc. I think some statistical evaluation could allow more concrete conclusions to be drawn. Furthermore, a feature-selection algorithm can be based on some statistics. I would suggest having a look at the recent BCI-research which provides many ideas on how to reduce multidimensional data.
5. The cited references in the introduction and discussion are reduced to a minimum. I would suggest doing some extensive research on movement-related EEG studies etc.

I have read this submission. I believe that I have an appropriate level of expertise to confirm that it is of an acceptable scientific standard, however I have significant reservations, as outlined above.

# Response

**Published:** 2014-04-24

**Authors:** Mario Manto

**Affiliations:** FNRS-Laboratoire de Neurologie Expérimentale, Hôpital Erasme, Université Libre de Bruxelles, Belgium

We thank the referee for their criticisms.

 

- *“Moreover, given the exhaustive analysis of EEG frequency ratios and EEG-MEG coherence, I would suggest applying some feature selection methods in order to overcome the problem of multidimensional data, which is hard to present and to interpret.”*

We have attempted to simplify the analysis, but unfortunately simplification would result in poorly informative findings. The field of BCI is highly complex and data often require an exhaustive approach in order to make BCIs really applicable to neurological patients. These patients are often heterogeneous in terms of mental imagery (see also the reply to Christoph M Michel) and a simple scheme could not be applicable to more than a single patient. This would mean that very few patients would benefit from BCI-based programs.

**Abstract**

- *“The sentence "A BCI-driven detection of voluntary movement is used to trigger FES in a closed-loop approach." sounds to me like you have used FES in this study. Perhaps it would be less misleading if you wrote this in the subjunctive.”*

The relevant paragraph of the abstract has been changed to the following:

*“The main purpose of this challenging project was to develop and validate a new treatment for upper limb tremor based on the combination of functional electrical stimulation (FES; which has been shown to reduce upper limb tremor) with a brain-computer interface (BCI); thus providing a BCI-driven detection of voluntary movement which can be used to trigger FES in a closed-loop approach.”*

Since this article’s publication, our global concept has now been validated by the MENRVA Group who used an affordable, BCI-controlled, wearable robot and electrical stimulation to assist subjects in drinking a glass of water. In this very recent study, the set-up required an EEG headset on the head of subjects along with FES electrodes on their arm. An embedded potentiometer was used to measure wrist rotation angle whereas an encoder in the elbow motor was used to quantify elbow angle. A gyroscope was used for the shoulder ( Looned *et al.,* 2014).

The general approach of the project has been presented earlier ( Rocon *et al.*, 2010).

**Introduction**

- *“In the introduction you emphasize the limitations of BCIs, which are based on conventional EEG analysis. Although I fully agree with you, this is a rather unspecific statement. Does this mean that "conventional" EEG analysis is less reliable for BCI-performance than "high-tech" EEG-analysis? Although we both know that this is not the case (BCIs are fed with highly sophisticated markers of the EEG and still do not perform at 100%), the sentence could be understood in this way. Therefore, I would suggest that you specify that you mean BCIs which ware based on EEG analysis only.”*

The word “only” has been added. As stated previously, recent studies point out that for some specific tasks a conventional affordable EEG can be used ( Looned *et al.,* 2014). We do not want to reduce the importance of EEG.

 

- *“I would like to make a further point on this: BCI users differ between each other in the way they can voluntarily modify their brain activity, and there is even an interindividual difference in the detectability of movement-related EEG-activity. Moreover, the fact that movement disorders often show up with brain atrophy or neuroplastic changes makes it difficult to generalize BCI markers that have been tested in a healthy population to patients with varying pathologies. I think this variance is a major reason why your multimodal processing could do so much better than a BCI-system that is solely based on EEG analysis.”*

We have added these sentences to the article.

**Methods**

- *“I don't think that the sentence "Patients and the experimental procedure were as detailed in the next sections." in the first paragraph of the Methods section is necessary (the reader sees that there is a section for patients etc.)”*

The sentence has been removed.

 

- *“Maybe in this first paragraph it would be nice to read that the different modules act during different time-windows”*

The paragraph has been changed to the following:

*“From each module, acting during different time-windows, (EEG, Kinematic and corticomuscular - described in detail in sections C-F) quality parameters (QPs) for the detection of the intentionality of movement or for the early detection of movement are extracted.”*

 

- *“Why did you prefer acoustically triggered movement over self-paced movement? How did you ensure that the acoustic signal did not influence the EEG signal which was used for detection of movement planning”*

This protocol did not generate artefacts as a consequence of the acoustic signal. Indeed, after the acoustic signal the subject first relaxes, then prepares the movement and subsequently executes it. This protocol provides a defined time window during which influences on EEG are minimized. Several groups in the world working with patients have observed that patients tend to execute automatic movements (probably with a smaller contribution of the cortical potential) when the task is self-paced. This is in contrast with experimental findings in control subjects ( Niazi *et al.*, 2012). Providing an acoustic cue is particularly interesting in Parkinson’s disease ( Lohnes and Earhart, 2011). It is plausible that a visual or somatosensory cue could also be used ( Rochester *et al., *2007).

 

- *“Why did you restrict your EEG-setup to centrally positioned electrodes? Movement planning involves frontal regions (e.g. electrodes F3, F4, F7, F8) and it is highly likely that you get a better prediction of movement if you include signals from these positions”.*

Our ultimate aim is to propose a wearable EEG cap with a few channels, in order to reach the goal of a BCI which could be used in daily life (outside the laboratory and the clinic) by neurological patients. Using 14 electrodes, we have detected the ERD/ERS phenomenon. The conventional caps which include the prefrontal electrodes require an important signal processing step, because of the artefacts generated - in particular by the frontalis muscle. This muscle often contaminates the EEG traces in neurological patients. Note McFarland *et al.* (2000)’s study which focused on the central areas of the brain for the principal component analysis related to motor imagery and movement. Schröder *et al. *(2003) have shown that choosing physiologically motivated channels improves classification accuracy when compared to all-channels. The choice of a subset of EEG channels is one of the selection features for the classification of EEG signals in BCI systems in order to avoid dealing with high dimensional and noisy data.

 

- *“How did you avoid blinking during the recordings? If you tell the participants that they should not blink, they might concentrate on this instruction instead on the task, making EEG-data less valid. In addition, participants who are told not to blink usually blink more frequently than if you would not mention blinks”*

Patients were told not to blink only in the period after the acoustic signal until the end of the movement. They were free to blink or swallow after the end of the movement until the successive acoustic signal.  Therefore, we did not encounter difficulties related to blinking prevention.

- *“* *I did not understand the reason why you only used three colours for the lines in figure 2. The reader can distinguish the three cases but not which sensor is represented by what line. Moreover, the first blue line, entitled "Mouvement..." should be entitled Movement... and should be a dashed line as indicated below the figure. Are the three cases three different sensors, three patients or three movements?* *”*

We have modified Figure 2.

 

- “ *You write "upsampled EEG data..." and indicate that there were 256 samples - is this one second? Does an overlap of 250 in the time domain refer to the original sampling rate or to the upsampled data, resulting in 250ms? Did I get this right; the Hamming window was 1 sec but overlapped with 250ms?* *”*

Data were upsampled from 256 samples to 1000/sec. Subsequently, signal processing is applied to these upsampled data.

 

- *“* *I do not exactly understand how you realized that the pre-movement period lasted for 2 sec, but participants performed the task following the acoustic signal. Were participants instructed to wait 2 sec? Or is this the average time the participants took until they performed the task?”*

The acoustic signals indicate the beginning of the test. After hearing the signal, the patients relaxed their face (no more blinking, swallowing....), prepared by mental imagery, and then executed the movement. In the study of Defebvre *et al. *(1999) desynchronization of EEG was recorded 2 s before to 0.5 s after voluntary wrist flexions from 11 leads covering the primary sensorimotor cortex (central), supplementary motor area (frontocentral) and parietal cortex (parietocentral). This has been added to the Methods.

 

- *“* *I did not understand what you intended by the (8 Hz, 9 Hz, ... 12 Hz) after the first interval.* *”*

These are frequencies of interest that are used for the analysis.

 

- *“Possibly because this is the first time I have read about this very interesting method of correlating brain and muscle signals - I did not fully understand the segmentation/processing of the data. You segmented data into epochs of 200ms and calculated the FFT on this window - what then does the "window of 8 samples" mean? Moreover, how did you determine the optimal window length? I see that the epochs vary between the modules.”*

This is a conventional method to calculate the FFT on epochs of data. According to the module used, the window may vary because the window is adapted to obtain the more relevant spectral information (see the details in McNames (2013)). The values used are based on our experience with signal processing in movement disorders.

**Results **

- *“Why do you refer to reference no. 8 when you report your results? Aren't these your present results?”*

The present study provides a detailed analysis and novel data as compared to the previous reference, which corresponded to a conference presentation. In addition, the present article includes the simulation study which is novel and was not published earlier.

 

- *“The central areas showed the highest values of QPs - indeed, you used only a central montage?”*

We represent here the QPs that reached a good value of accuracy.

 

- *“* *You talk about means - obviously, you may indicate averages over patients, but did you also average over electrodes?* *”*

No, we did not average the traces over the electrodes. This results in a lower spatial resolution, especially in neurological patients with focal brain lesions or disorders. We have observed that individual channels provide more meaningful information as compared to averages for the task of finger-to-nose. Other studies have been performed in the framework of this project and have not shown a superiority of averages (they rather showed a lower performance when all the channels are averaged).

 

- *“(Fig. 3)* *What electrode positions are used to build this figure?* *”*

These data correspond to the central area of the brain. This has been added to the revised article.

 

- *“(Fig. 4) The vertical dashed lines seem to occur periodically (each 20ms) with a certain time-distance but not necessarily on the highest peaks - do you have an interpretation/explanation for this phenomenon?”*

This is well known for the cortico-muscular coherence. This is why some groups use a flexible window in their software to extract the best cortico-muscular coherence. There are several explanations: the variability in the preparation of movement, the variability in the corticospinal tract command from trial-to-trial, the variability in the muscle contraction from trial-to-trial and the electro-mechanical delay in muscle contraction (see Jenkins, Palmer and Cramer (2013) and Howatson *et al.* (2009)).

 

- *“(Fig. 5) With what rationale were the 2 patients and 3 trials chosen?”*

These patients exhibited reproducible low values for the cortico-muscular coherence, by contrast to reproducible high values for the QP. This highlights the importance of our approach. This has been added to the revised article.

 

- *“(Fig. 6) It would be helpful to rescale the x-axis, so that the beginning of movement is time 0 and then indicate steps of +-250ms.”*

It might be interesting to keep the same presentation in order to highlight the prediction phase from the post-movement period.

 

- *“* *(Fig. 9) What is the EEG/CorticoMuscular probability based on - averages across channels/channel combinations or one single channel/combination (the best one?)?”*

The combinations of EEG/EMG were done for each electrode. Averages across channels decrease the values obtained, and therefore reduce the strength of the study. This is in agreement with the current wearable EEG caps, which include a few selected channels only (and this is also a better approach in terms of ergonomics and aesthetics).   

 

- *“(Fig 14) There are not only continuous and dotted lines but also external lines with "big dots" - is this the range (min-max)?”*

External lines with larger dots indicate the 95% confidence interval. This has been added in the legend of the figure in the revised article.

**Supplementary data**

- *"What do the rows in e.g. 001FN03.mat_EE...csv mean? Are these samples?"*

Yes, these are samples. The referee and the reader can use the data to extract QPs and compare with other methods they might develop in the future.

**Discussion**

- *“You state that QP values greater than 90% were observed in some of the runs...That is, QP was calculated for each run? This is not clear from the methods section.”*

QPs were calculated for each movement executed by the patients. One run contains several movements. QPs extracted showed good accuracy (fixed for values higher that 70%), with some QPs which were extremely good (accuracy greater than 90%). The methods section has been modified accordingly in the revised version of the article.

 

- *“Why are EEG recordings more complex when tremor is generated in nuclei deep in the brain?”*

Because EEG activity is mainly driven by the cortical brain electrical activity, which is detectable through the scalp. Activities generated in depth, for instance in basal ganglia (putamen, caudate nucleus…), are very weakly recorded with conventional EEG electrodes. They require the use of invasive deep brain electrodes. The same observation can be put forward for tremor related to lacunar stroke.  

 

- *“* *In the discussion of kinematic data it seems to me that you discuss the background generally without referring to your own results. You should rather discuss why the y axis alone is superior to x axis or a combination of the two.* *”*

We have added one sentence explaining that there is very often a so-called dominant axis which provides the most meaningful information. For instance, in cases of cerebellar kinetic tremor, the vertical axis perpendicular to the direction of motion is the most meaningful (relevant for kinetic tremor).

 

- *“* *Do probability trees really show the best association of parameters? In a subsequent sentence you write that all possible combinations need to be tested. To me probability trees as used in this paper seem to give the impression of how complex the problem is, but they do not answer the question: Which feature/combination of features leads to highest detection rates? This problem is solved by feature selection algorithms (see general remark 2).* *”*

The best association of QPs is a case by case decision. We confirm that the probability trees can be used to extract the best combination. However, these probability trees have been built for a given task and it is very likely that the ultimate choice will rely on the selection of the best probability trees related to a given number of selected tasks representative for daily life. A specific study should address this very interesting but very complex question.

 

- *“I am not an FES-expert, but wonder if there is any literature about the instance of time when FES has to be applied in order to make a movement free of tremor. Should it be applied at movement onset, or before the movement is carried out, i.e., in the planning phase? It probably depends on each individual patient, since tremor can occurr pre- movement or during movement. Thus, the kind of FES should be different for each patient and similarly, depending on the type of application, the EEG-part may or may not be useful. Also if planning the action induced the tremor, detection of movement intention based on EEG could be too late?”*

Muscular FES induces mainly a peripheral suppression of tremor, since electrical stimulation exerts its effect on the activities of the agonist and antagonist muscles of the trembling limb, rather than on the central sources of tremor.  Our aim is to trigger the electrical stimulator on the basis of an early detection of the intention of movement. FES appears as a viable option to suppress the different forms of tremor. Several articles have been published on this topic. For instance, our group has now demonstrated that FES is effective to block physiological tremor - Grimaldi, Fernandez and Manto (2013). The intensity of stimulation and the pattern of stimulation can be adapted as a function of the percentage of tremor reduction.

 

- *“There is no discussion about the simulation results.”*

The following sentences have been added to the revised version of the article:

*“Results obtained with the simulation study provide useful information about EEG QP in order to select patients more effectively for a BCI-based treatment, including rehabilitation. The simulation demonstrates the relationship between the threshold and the QP. Future studies could take advantage of these findings to select the best neurological candidates on the basis of the ERD/ERS for BCI-based management."*

**Conclusion**

- *“* *In the conclusion it seems that the QP is an EEG-specific value which can be complemented by values from other modules (kinematic...) - instead, the QP is a value for estimating the performance in detecting movement and can be applied to each modality. But is this really a main conclusion? I think the conclusion should be that the EEG can be supplemented by other modalities. However, the extent to which the detection rate of the EEG can be supplemented, i.e., how much the QP can be increased by combining several modalities, has not been evaluated.* *”*

The paragraph has been changed to the following:

*“We propose that the EEG QP can be complemented by the QPs extracted from the cortico-muscular coherence and the QPs obtained by the analysis of the changes in the kinematic signals, which occur prior to the voluntary movements. We suggest a fusion of the QP parameters in order to increase the likelihood to detect the intentionality of movement. The analysis of the corticomuscular coherence shows that this parameter alone cannot be used to predict voluntary motion and be implemented in a BCI”*.

 Some studies focusing only on corticomuscular coherence have failed.

 

- *“The last minus one sentence: The term classifiers comes out of the blue. This technique deserves some place in the discussion (as suggested in general remark 2).* ”

The word “ *classifier”* has been changed to:

*“classification algorithms for BCI system in order to extract EEG patterns related to a cognitive or motor status”*.

The following reference has been added: Pfurtscheller *et al.* (1996).

 

- *“*Last sentence: The approach has firstly to be implemented - the present manuscript is a pre-study. The next step is to integrate all of the modalities. Then you could evaluate the approach on a large sample. *”*

The sentence has been changed to:

*“Our approach will have to be tested in a large sample of patients in the future, in order to demonstrate its real clinical usefulness in daily practice. We propose to select a larger group of neurological patients to confirm the strength of the multimodal prediction.” *in the revised version of the article.

**General remarks**

- *“* *You use BCI in terms of a control unit. BCI is an interface between brain and computer. As such, your system would be a multimodal control unit, including also an EEG-module like often used for BCIs, but also body modules to control a stimulation unit. I think the term BCI is not the best choice here, since it does not take into consideration the EMG module etc.* *”*

The terminology is used in the literature, but we understand the point made by the referee. In the revised version of the Discussion, we have introduced the concept of a *“multimodal control unit”:*

*“In theory, BCI is an interface between brain and computer. As such, our system would be a multimodal control unit, including also an EEG-module like often used for BCIs, but also body modules to control a stimulation unit.”*  

 

- *“* *The way such a problem would be solved by the current BCI-community is a classification by use of machine learning techniques i.e., you apply some feature selection algorithm and train your machine in discriminating movements based on the multimodal input. These two steps could further be included in one step e.g. by use of random forests. By doing so you evaluate the performance of your modules and find out which ones contribute most to a high detection rate - separately for each patient (thus, you take into account interindividual variability). I would at least include this option in the discussion.*”

This is a great idea. We have added the following sentence to the Discussion:

*“Future works could* *apply some feature selection algorithm and train the* *multimodal control unit* *in discriminating movements based on the multimodal input. These two steps could further be included in one step e.g. by use of random forests. By doing so, the performance of the modules would be evaluated to find out which ones contribute most to a high detection rate. This would be done separately for each patient, thus taking into account the inter-individual variability.”*

 

- *“The way such a problem would be solved by the current BCI-community is a classification by use of machine learning techniques i.e., you apply some feature selection algorithm and train your machine in discriminating movements based on the multimodal input. These two steps could further be included in one step e.g. by use of random forests. By doing so you evaluate the performance of your modules and find out which ones contribute most to a high detection rate - separately for each patient (thus, you take into account interindividual variability). I would at least include this option in the discussion.”*

We have added the following sentence to the Conclusion:

“ *The present study opens the door for future studies in terms of how to increase EEG-based detection of movement intention by incorporating information from multiple modules.”*

We feel that we respond partially to this critical question, for instance by showing that a cortico-muscular module cannot provide a good evaluation of the intentionality of movement (some groups still try to rely on this parameter to detect the preparation of movement and to manipulate an effector).

 

- *“The presentation of the results is rather descriptive, the authors report QP, SDs etc. but no statistical test is applied. It would have been interesting if the QPs differ between modalities, within modalities with respect to electrode locations etc. I think some statistical evaluation could allow more concrete conclusions to be drawn. Furthermore, a feature-selection algorithm can be based on some statistics. I would suggest having a look at the recent BCI-research which provides many ideas on how to reduce multidimensional data.”*

We have added the references below and have included the following sentence in the Discussion:

*“Techniques of multichannel EEG compression, phase congruency and graphical representations aiming at a reduction of multidimensional data have been proposed [Gasser and Möcks, 1983;* *Logesparan* *and Rodriguez-Villegas, 2010; Dauwels et al. 2013]. However, no technique has been widely accepted so far.”*

Gasser and Möcks (1983)

Logesparan and Rodriguez-Villegas (2010)

Dauwels *et al.* (2013)

The following references have been added:

Rektor, Sochůrková and Bocková (2006)

Shibasaki and Hallett (2006)

# Peer review: Referee response for version 1

**DOI:** 10.5256/f1000research.3288.r2881

**Published:** 2014-01-03

**Authors:** Christoph M Michel

**Affiliations:** 1 Functional Brain Mapping Laboratory, Department of Neuroscience, University of Geneva, Geneva, Switzerland

The manuscript evaluates the use of a combination of neurophysiological signals to detect movement intention in tremor patients. Multichannel scalp EEG, multi-array MEG and kinematic sensors were jointly analyzed in a trigger-initiated finger-to-nose movement task. The aim was to optimize detection of movement initiation. Four patients with upper limb tremor were studied.

The paper is technically sound and the analysis methods are clear. However, some of the analyzed parameters are irrelevant, redundant or highly correlated, making it difficult to understand why they were all performed separately. I have the following major comments:

 

1. EEG analysis of Beta-Alpha ratio was analyzed in 105 different combinations of frequency bands that were highly overlapping. The rational for this fine distinction of different frequency bands is not very clear given the existing (but not cited) literature on event-related synchronization/desynchronization (work by Pfurtscheller, for example). While not explicitly stated, I guess that these 105 combinations were done for each electrode. Studying the existing literature would have allowed the authors to restrict to the known electrodes and frequency-bands of interest.
2. EEG-MEG coherence analysis: each EEG channel is correlated with one MEG channel. Figure 4 shows very similar results for each electrode. This is not surprising given that each EEG electrode measures part of the activity of the same generators in the brain. Volume conductance makes the different EEG signals highly correlated when referred to a common reference and thus they are all correlated with the MEG waveform.
3. It is not clear whether the results are specific to tremor patients - a control group is missing.
4. The results are not clear. One would have expected a conclusion that informs the reader which of the multiple parameters seem to be most promising. The presented probability trees are very abstract and do not allow one to make a conclusion on specific parameters.
5. The Table at the end of the manuscript is not clear. What do the different numbers represent?
6. The abstract should not be a verbatim copy of the Introduction.
7. A better description of the literature on ERD in BCI is needed in the Introduction. Important work is missing.

I have read this submission. I believe that I have an appropriate level of expertise to confirm that it is of an acceptable scientific standard, however I have significant reservations, as outlined above.

# Response

**Published:** 2014-04-24

**Authors:** Mario Manto

**Affiliations:** FNRS-Laboratoire de Neurologie Expérimentale, Hôpital Erasme, Université Libre de Bruxelles, Belgium

We thank the referee for their criticisms.

1. *“EEG analysis of Beta-Alpha ratio was analyzed in 105 different combinations of frequency bands that were highly overlapping. The rational for this fine distinction of different frequency bands is not very clear given the existing (but not cited) literature on event-related synchronization/desynchronization (work by Pfurtscheller, for example). While not explicitly stated, I guess that these 105 combinations were done for each electrode. Studying the existing literature would have allowed the authors to restrict to the known electrodes and frequency-bands of interest.”*

We have added the following sentences in the Discussion:

*“Our protocol in neurological patients with tremor differs from those in the literature, hence our study on the multiple combinations of frequency bands. When a neurological patient with tremor is seated and assessed, he/she may exhibit a tremor of the head and trunk. This tremor may be pretty stable or rather intermittent. There may even be an overlap with the main frequencies of the EEG signal, for instance in the alpha band (a rapid head tremor may be found). Therefore, we decided to have a close look to each of these bands. For instance, we have seen patients with cerebellar disorders and orthostatic tremor in whom the sub-band 8-10 Hz was much less informative as compared with the sub-band 10-12 Hz. We would like to point out that in the study of Pfurtscheller et al. on single-trial classification of EEG and imagination (Neuroimage. 2006;31(1):153-9), the frequency of the most reactive components was 11.7 +/- 0.4 Hz (mean +/- SD).  The SD was thus small. Although the desynchronized components were centered at 10.9 Hz +/- 0.9 Hz, the synchronized components were narrow-banded, with higher frequencies at 12.0 Hz +/- 1.0 Hz. We agree with the authors that the classification of single EEG trials improves when ERD and ERS patterns are combined for multiple tasks. We aim to pursue the use of narrow bands of frequency in multiple tasks.”*

2. *“EEG-MEG coherence analysis: each EEG channel is correlated with one MEG channel. Figure 4 shows very similar results for each electrode. This is not surprising given that each EEG electrode measures part of the activity of the same generators in the brain. Volume conductance makes the different EEG signals highly correlated when referred to a common reference and thus they are all correlated with the MEG waveform.”*

The combinations of EEG/EMG were done for each electrode. We agree that in theory the volume conductance effect and the common generators could impact on the coherence analysis in healthy subjects. The rationale is the following: EEG signals taken from nearby areas may be very different in patients with brain lesions (the density of MEG channels is also much higher as compared to our set-up). This is due to structural lesions in the brain. These MRI images (T2-weighted axial sections) illustrate the subcortical lesions in a patient with Parkinsonism of vascular origin. The lesions disrupt the subcortical tracts of the periventricular white matter. The attempt to translate data obtained in healthy subjects cannot be efficient. In these patients, a case-by-case BCI scenario is required. We want also to point out that fMRI studies show an activation of selected areas of the homunculus during limb movements. Hence also the selection of the central area for the EEG channels (see the reply to Yvonne Hoeller). Several groups working on BCIs are now attempting to focus on the EEG signals located nearby the pre-central sulcus. We would like to stress that our patients do exhibit tremor (including of the head-trunk), unlike control subjects. The algorithm suggested for tremor suppression requires tremor. It might be interesting to investigate patients showing tremor likely induced by a disruption of the peripheral nervous system, and to compare the results with those obtained in patients showing brain lesions.

This figure illustrates an example of the results of ERD/ERS in a patient with post-traumatic tremor (multiple brain lesions) pressing on a force transducer during a pinch task of the right hand. The patient is seated in front of a computer: (a) baseline measurement (rest) during 2 seconds, followed by (b) the patient prepares himself by mental imagery for 4 seconds, and (c) the patient presses a force transducer during 2 seconds. The channels C3-Cz and FC3-Cz are compared. The relative results for the main sub-bands (delta, theta, alpha, beta) are shown. Note the clear difference between the ratio beta/alpha. The ERD/ERS is much stronger for C3-Cz as compared to FC3-Fz.

3. *“It is not clear whether the results are specific to tremor patients - a control group is missing.”*

Our project focused on tremor patients and not on healthy subjects. This has been addressed by other partners of the project. We would like to point out that the attempt to translate findings in healthy subjects to neurological patients with brain lesions is very likely to be poorly productive. See also the reply to the previous query.

*“The results are not clear. One would have expected a conclusion that informs the reader which of the multiple parameters seem to be most promising. The presented probability trees are very abstract and do not allow one to make a conclusion on specific parameters.”*

We have modified the Discussion as follows:

*“Our data provide a ground for the concept of multimodal approach developed for the early detection of the intentionality of movement. The presented probability trees are general schemes. A case-by-case analysis is required. In order to provide the most possible accurate BCI-driven FES system, each subject needs to be studied in order to define the best combination of QPs. For instance the kinematic QPs may be more efficient than the EEG QPs in a given patient (as it may happen when ERD/ERS is not stronger enough to be detected). The system would take into account these features. By analysing a larger group of patients, we might identify subgroups of patients on the basis of the results of the probability trees. In other words, the probability trees would be used as an eligibility procedure to multimodal BCI-driven treatments in neurological patients with tremor”.*

- *“The Table at the end of the manuscript is not clear. What do the different numbers represent?”*

Data from experiments have been included, so that the referee or the reader can compare them with his/her own software.

- *“The abstract should not be a verbatim copy of the Introduction.”*

The introduction has been re-written.

*4.  “A better description of the literature on ERD in BCI is needed in the Introduction. Important work is missing.”*

The following references have been added:

· Pfurtscheller and Lopes da Silva (1999)

· Pfurtscheller *et al. *(2006)

· Birbaumer *et al. *(2006)
